# Motor Expertise Combines Lower Task Engagement with Enhanced Error Sensitivity

**DOI:** 10.1007/s12311-026-02100-4

**Published:** 2026-11-04

**Authors:** Pavel Filip, Lenka Marková, Cecile Gallea, Stéphane Lehéricy, Ovidiu V. Lungu, Shalom Michaeli, Silvia Mangia, Viktor Weiss, Martin Bareš, Lubomír Vojtíšek

**Affiliations:** 1 Department of Neurology, Charles University, First Faculty of Medicine and General University Hospital, Prague, Czech Republic; 2 Department of Cybernetics, Czech Technical University in Prague, Prague, Czech Republic; 3 Center for Magnetic Resonance Research (CMRR), University of Minnesota, Minneapolis, MN USA; 4 Intensive Care, Rescue and Pain Medicine, Canton Hospital St.Gallen Clinic for Anaesthesiology, St. Gallen, Switzerland; 5 Institut du Cerveau–Paris Brain Institute–ICM, Inserm, CNRS, Sorbonne Université, 75651 Paris, France; 6 Department of Psychiatry and Addictology, University of Montreal, Montreal, Québec Canada; 7 First Department of Neurology, Faculty of Medicine, Masaryk University and St. Anne’s Teaching Hospital, Brno, Czech Republic; 8 Central European Institute of Technology, Masaryk University, Brno, Czech Republic

**Keywords:** Motor learning, Predictive control, Error correction, Functional MRI, Network reorganisation, Quantitative MRI, Microstructural plasticity

## Abstract

Skilled motor behaviour relies on complex predictive control and error processing. Despite its indispensable position in our everyday lives, neurobiological mechanisms of motor skill acquisition remain poorly understood. The presented study evaluates the functional dynamics and microstructural substrate of expertise-related reorganisation in predictive timing circuits using multimodal functional and quantitative magnetic resonance imaging (MRI) data. Forty-one baseball/softball players, representing a natural model of optimised motor timing programs, were compared against forty-six non-athletic controls using a structural connectome-informed hierarchical Bayesian framework jointly modelling task-based functional MRI dynamics of feedforward predictive control and error-driven modulation. Expertise-related microstructural differences were then characterised using neurite orientation dispersion and density imaging and adiabatic T₁ρ mapping. In non-athletic controls, predictive control was found to engage a distributed cortico-subcortical sensorimotor, visuospatial, attentional and frontoparietal loops, while higher-order associative systems were relatively down-regulated. However, quantitative deviation from the task optimum was associated with lower response across this predictive network. Expertise was characterised by higher behavioural performance but also by attenuated prediction-related functional engagement alongside enhanced error-related modulation. Furthermore, neurite density and orientation dispersion indices were higher in experts across distributed cortical networks, while the cerebellum exhibited a divergent profile characterised by higher neurite density, lower orientation dispersion and faster T₁ρ relaxation. These findings indicate that expertise involves lower task engagement within an established architecture despite higher behavioural performance, together with enhanced error sensitivity and distributed microstructural differences. This pattern is consistent with more efficient neural dynamics complemented with underlying tissue alterations.

**Supplementary Information:**

The online version contains supplementary material available at https://doi.org/10.1007/s12311-026-02100-4.

## Introduction

Motor program optimisation stands at the core of our daily experience, enabling rapid, reliable and precise behaviour with minimal conscious supervision. It engages a distributed network system encompassing the cerebellum, basal ganglia, motor cortex and several brainstem structures [1]. Despite its central role, the mechanisms of adaptive motor regulation underlying motor skill acquisition and their optimisation to reduce the reliance on cognitive control, with its inherently limited capacity, remain poorly understood.

Converging theoretical and empirical work increasingly supports the view of brain architecture and both motor and non-motor programs as fundamentally predictive in nature [2, 3]. Several theoretical concepts propose predictive control as a distributed process involving recurrent interactions across distributed cortical, subcortical and cerebellar networks [4], with varying contributions of individual nodes across specific domains [5–8]. Within this context, cerebellum has been implied as a major substrate responsible for fast, high-fidelity predictive control at short time scales complementing cortico-subcortical networks [1, 9].

This framework points to a complex, fluid processing environment enabling skill acquisition and further predictive control optimisation. Learning may then be conceptualised as delicate re-balancing of increasing accuracy of internal prediction and management of deviations from desired or expected outcomes. However, analytical separation of these conceptual counterparts using available measurement methods is non-trivial. Task functional MRI (fMRI) provides a neurobiologically plausible, yet indirect window into internal model use. Paradigms focusing specifically on predictive timing [10, 11] offer a viable opportunity to dissociate prediction-related neural programs from motor execution per se. Participants generate and update estimates of future state, engaging cortico-cerebellar and cortico-basal ganglia circuits, which allows for the observation of the complex prediction-related functional patterns even within extensive in-scanner movement limitations.

The presented study utilises this paradigm in a population where long-term, in-depth experience with predictive control is a core feature. Baseballers are a natural model of expertise-driven program optimisation and have been previously shown to outperform non-athletic controls in movement-based prediction [12]. In the current mechanistic extension of our previous efforts, the overarching aim was to dissociate the effect of optimisation into its two major presumed components – tonic control processes responsible for predictive task execution and transient error-driven updates mitigating deviations from an internal optimum. We hypothesised that predictive control optimisation would be associated not only with higher effectiveness – more accurate internal predictions with lower need for deviation correction– but also with higher efficiency – expressed as hyper-optimisation of predictive motor networks where greater behavioural performance is achieved with smaller fMRI signature. Furthermore, we tested whether program optimisation is also associated with underlying tissue reconfiguration utilising indirect microstructural proxies of dendritic complexity and macromolecular content derived from neurite orientation dispersion and density imaging (NODDI) [13] and adiabatic longitudinal relaxation [14], respectively.

## Methods

43 baseball/softball players (hereinafter experts) (12 females – softball players, 31 males – baseball players; median age 24, range 17–37) and 46 non-athletic controls (15 females, median age 24.5 [18–39]) were enrolled into this study. All experts were selected from the Czech Baseball National Team or high-level domestic league, with at least 10 years of competitive experience. All participants were right-handed according to the Edinburgh Handedness Inventory and reported no history of neurological and/or psychiatric complaints or standard MRI contraindications. Every participant provided their written consent in accordance with the Declaration of Helsinki. The study protocol was approved by the Ethics Committee of the University Hospital of St. Anne, Czech Republic (approval code 38 V/2018).

### Experimental Task

**Fig. 1:**
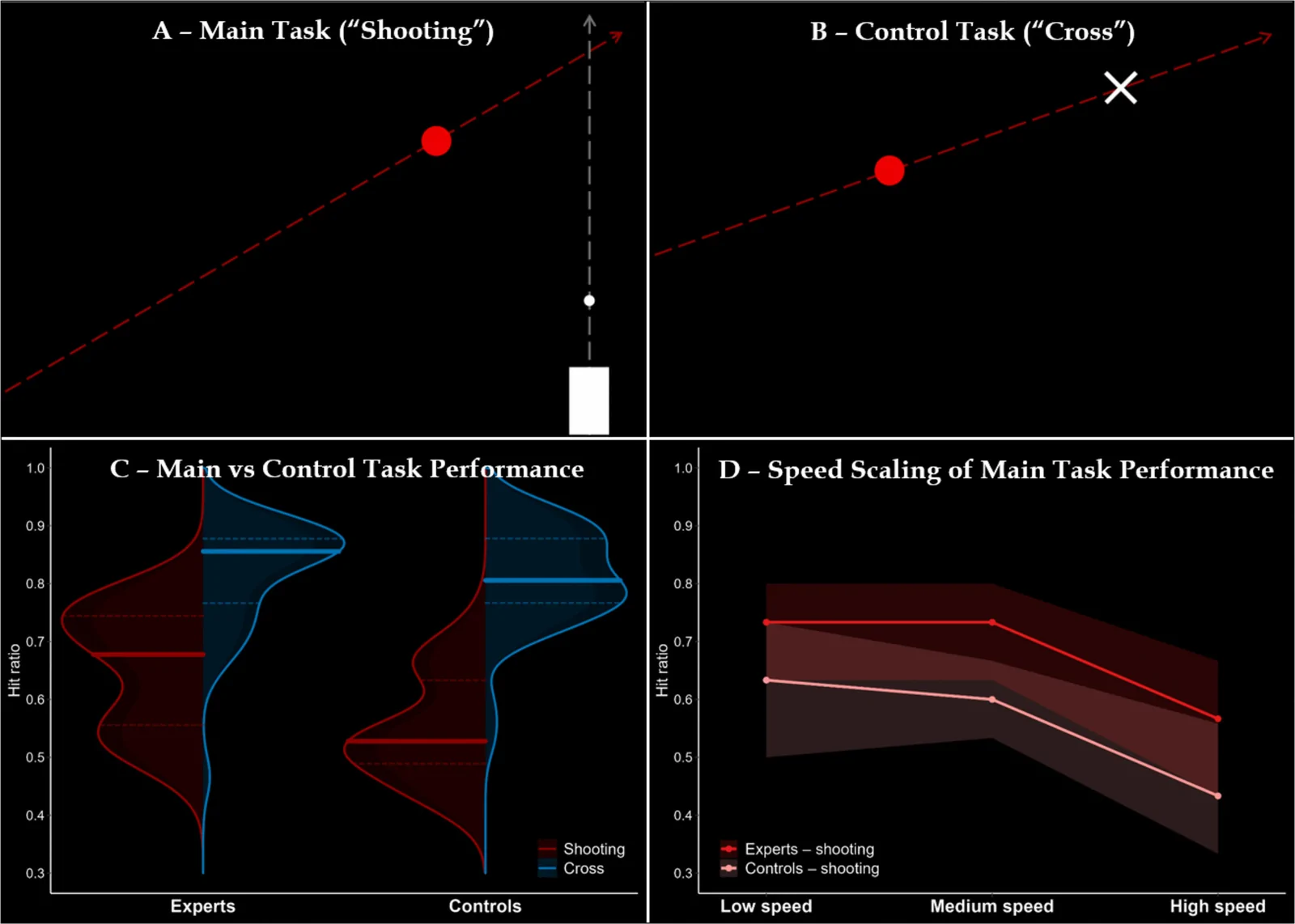
Behavioural task design and group performance. **A** Schematic of the main “shooting” predictive timing task. A moving target (red ball) flew from the left side of the screen under random angles into the right upper corner to be intercepted by a projectile fired by the participant vertically from the right lower corner. Dashed arrows indicating trajectories for visualisation purposes were not present on the screen. **B** Schematic of the control “cross” condition, where participants responded when the ball reached the fixed cross positioned randomly over the second half of the target trajectory. **C** Main vs control task performance shown as split violin plots of proportional hit rate, aggregated per participant across sessions and speed levels. Shooting (red, left half) and Cross (blue, right half) shown separately for Experts and Controls. Solid horizontal lines indicate medians; dashed lines indicate quartiles. **D** Speed scaling of main task performance. Group-wise median hit rate across low/medium/high speed levels with shaded interquartile range. No inferential statistics provided, see [12] for detailed analyses. Full numerical results underlying the figures are provided in the Supplementary Dataset

Participants performed a previously published motor timing task [10]. In the main time-estimation condition (“shooting”), participants pressed a button to launch a vertically moving projectile to intercept a circular target moving from the left side of the screen at one of three possible speeds (Fig. 1A). The deliberately low velocity of the projectile required anticipatory timing – button had to be pressed before the target reached the fixed interception zone in the upper right corner, to account for the time needed by the projectile to also reach the interception area. The diagonal angles of the target trajectory were selected randomly to limit learning. A control condition (“cross”) matched visual input characteristics and motor output of the main condition but lacked the predictive timing aspect since button press was required when the target entered a zone explicitly marked with a cross (Fig. 1B). Visual feedback indicated hit or miss after each trial in the form of a green or red fixation point, respectively, presented in the centre of the screen for about 5 s (with random jitter of ± 1 s) after each trial. Trials were organised into blocks of 10 shooting trials followed by 10 cross trials, repeated three times per session, with target speed randomised to provide 10 high/medium/low-speed trials per condition (shooting/cross). Participants completed a brief training session during the acquisition of the preceding structural scans.

### MRI Data Acquisition and Processing

MRI data was acquired using a 3 Tesla Siemens MAGNETOM Prisma with 64-channel head/neck coil at the Central European Institute of Technology in Brno, Czech Republic, with the following protocol covering the whole brain including the brainstem and cerebellum (except T₁ρ, see below):

- T₁-weighted (MPRAGE): isotropic 0.8 mm^3^; repetition time (TR)/echo time (TE)/inversion time (TI) = 2,300/2.28/1,000 ms; flip angle (FA) 8°; GRAPPA 3.
- T₂-weighted (SPACE): isotropic 0.8 mm^3^; TR/TE = 3,200/409 ms; GRAPPA 2.
- Diffusion-Weighted Imaging (DWI): isotropic 1.5 mm^3^; TR/TE = 3,230/89.2 ms, effective echo spacing 0.69 ms; multiband (MB) acceleration factor 4; two non-zero shells b = 1,500/3,000 s.mm^−2^, 93 directions, 7 interleaved b0 volumes; acquired twice with reversed phase-encoding (antero-posterior [AP], and postero-anterior [PA]) [15].
- fMRI (blood-oxygenation-level–dependent (BOLD) gradient-recalled echo echo-planar imaging): 3 sessions, isotropic 2.0 mm^3^; TR/TE = 820/35.2 ms; FA 45°; MB 4, PA phase encoding; 590 volumes per session; Spin-echo fieldmaps (AP/PA) were acquired for distortion correction.
- Adiabatic T₁ρ: acquired only over a slab covering the cerebellum and basal ganglia; in-plane resolution 1.0 × 1.0 mm^2^, slice thickness 3 mm, 23 slices in total; TR/TE = 2,000/2.33 ms; GRAPPA 3; AFP HS4 pulses (R = 10, duration 6 ms; 0–16 pulses [0/4/8/12/16; 0–96 ms] phase-cycled by MLEV-4; bandwidth 1.3 kHz; ω₁max/(2π) = 800 Hz) [16, 17].

The processing pipeline was based on the Human Connectome Project (HCP) minimal preprocessing pipeline, including the optional correction of gradient non-linearity and susceptibility-related B_0_-field distortions [18] and the subsequent ICA + FIX denoising of fMRI data [19]. DWI data processing implemented upstream denoising based on random matrix theory with Marchenko-Pastur distribution modelling [20] utilising the optimised patch-based singular value shrinkage method [21], the removal of Gibbs ringing artefacts via local subvoxel shifts [22], followed by the HCP minimal preprocessing pipeline architecture with mitigation of eddy-current distortions extended by slice-to-volume motion correction [23], outlier replacement [24] and susceptibility-by-movement interaction correction [25]. A three-tissue response function was estimated and used for multi-shell multi-tissue constrained spherical deconvolution to derive fibre orientation distributions [26], followed by multi-tissue log-domain intensity normalisation. Anatomically constrained probabilistic tractography (FreeSurfer-informed) was performed using iFOD2 with dynamic seeding to generate 10 million streamlines [27], which were subsequently reweighted using SIFT2 to obtain quantitative connectivity estimates [28].

Microstructural parameter estimation fitted the NODDI-Bingham model to DWI data using Bayesian inference framework with Markov chain Monte Carlo sampling [29], with 5,000 burn-in iterations followed by 25,000 iterations with thinning by a factor of 25, yielding voxel-wise neurite density index (NDI) and orientation dispersion index (ODI). NDI and ODI maps were then sampled onto subject-specific mid-thickness cortical surface using cortical-normal interpolation with grey matter weighting to minimise partial volume effects. Mapping of T_1_ρ relaxation time constant was preceded by 3D rigid-body realignment of all volumes to the first scan (mutual information cost function with trilinear interpolation, with subsequent sinc interpolation refinement). Voxel-wise signal evolution was modelled using a two-parameter mono-exponential decay model to estimate the equilibrium signal intensity and the relaxation time constants with non-linear optimisation (Nelder-Mead simplex).

All processing outputs implemented a hybrid parcellation scheme combining HCP multimodal atlas (180 parcels per individually reconstructed hemispheric cortical surface) [30] and additional standard-space atlases: structural cerebellar [31], Oxford-GSK-Imanova striatal [32], Oxford thalamic connectivity [33], hypothalamic [34], ARAS [35], ATAG [36] and hippocampal atlas [37], collectively covering full extent of subcortical grey matter. Subcortical atlas masks were warped from the standard to individual T_1_-weighted space and rigidly aligned to DWI/T₁ρ mapping space in a single resampling step with trilinear interpolation. Regional values were calculated as parcel-level probability-weighted means. For fMRI data, HCP CIFTI cortical and standard-space output was utilised for parcellated timeseries extraction. For T₁ρ, only subcortical grey-matter parcels were considered due to limited acquisition coverage.

### Quality Control (QC)

Two participants (both in the expert group) failed to complete all the task fMRI acquisition and were excluded from further analysis. Moreover, T_1_ρ acquisition was not available for one further participant (expert) and, as a secondary metric, was imputed parcel-wise using hot-deck imputation: a donor participant was selected from the same group based on nearest-neighbour similarity in z-scored subcortical NDI and ODI profiles.

Subject-wise QC was performed in three tiers:

- Tier 1 (motion; hard criterion): sessions with pronounced motion spikes (95th-percentile framewise displacement [FD_95_] > ½ voxel: 1 mm for fMRI; 0.75 mm for DWI) were excluded.
- Tier 2 (multivariate stability): robust Mahalanobis distance based on the orthogonalised Gnanadesikan–Kettenring scatter matrix was calculated per session/modality from five stability metrics and flagged at χ^2^_(5; 0.999)_ = 20.52. For fMRI, these were FD_95_, 95^th^ percentile DVARS on residualised parcel timeseries, mean high-frequency power fraction (0.20–0.45 Hz relative to 0.01–0.45 Hz), 95^th^-percentile global-signal derivative, and mean fraction of extreme amplitudes (|robust z|> 5). For DWI, these were FD_95_, eddy slice-outlier fraction, reduced χ^2^ of the diffusion tensor fit, SIFT2 weight dispersion, and connectome edge density.
- Tier 3 (biological plausibility): sessions flagged in Tier 2 were excluded only if arbitrarily selected connectivity-based metrics not considered in the main analyses (homotopic symmetry and global connectivity) showed substantial within-cohort deviation (|robust z|> 5 in either metric).

Sessions failing Tier 1, or both Tier 2 and Tier 3, were to be excluded; only participants passing QC for DWI and all three task fMRI sessions were retained. These criteria did not flag any further subjects for exclusion. Ergo, the final analysis comprised 41 experts (group summary characteristics unchanged: 12 females, age 24 [17–37]) and 46 controls (15 females, age 24.5 [18–39]).

Parcel-wise QC was based on five technical fMRI measures (temporal signal-to-noise ratio, motion coupling, high-frequency contamination, spike rate, global signal leverage) averaged over the three task fMRI sessions and five technical DWI metrics (diffusion model misfit, thermal noise variance, streamline reduction ratio, absent-edge fraction, streamline weight entropy). Robust Mahalanobis distances were computed within subjects separately for cortical and subcortical parcels due to inherent hardware-related differences in signal characteristics and aggregated per parcel across subjects using median. 30 parcels exceeding D^2^ > χ^2^_(10; 0.999)_ = 29.59 (generally located in artefact-prone areas, see Supplementary Fig. 1) were excluded from all the following analyses.

### Activation Analysis

At the first level, task-related BOLD responses were modelled using event-related general linear model (GLM), with trial onsets set to the appearance of the target on the left side of the screen and durations to response times (button press). Condition- and speed-specific differences in the haemodynamic response were captured by partitioning into six condition cells distinguishing the main (shooting) and control (cross) tasks at each of the three target speeds. Trial-wise performance was quantified using timing error defined as the signed difference between the response time and calculated trial-specific optimum. Since temporal tolerance of successful hit varied with target speed, timing error was normalised within each speed level by robust estimate of its dispersion across trials. This speed-invariant measure of trial performance was again modelled separately for each condition and speed type (six regressors). Lastly, given the 10-trial block structure, two condition-specific nuisance regressors were constructed to capture block-wise variation, modelling block repetition separately for the shooting and cross task blocks (mean-centred within each session). All task and covariate regressors were subsequently convolved with the SPM canonical haemodynamic response function (30 s kernel) and sampled at fMRI acquisition times to form a volume-level design matrix. The resulting design matrices exhibited low collinearity, with variance inflation factors for all regressors below 2.

Further subject-level processing of parcellated fMRI timeseries implemented spatial Bayesian general linear modelling approach using integrated nested Laplacian approximations (INLA) [38] instead of standard mass-univariate analyses. This approach enabled joint estimation of task effects with explicit modelling of inter-parcel spatial dependence derived from subject-specific structural connectomes, providing higher robustness to noise and full posterior uncertainty quantification for downstream group analyses. Following the removal of nuisance variance attributable to head motion (six rigid-body parameters with temporal derivatives) and low-order drifts (linear and quadratic trends) from both the task regressors and parcellated fMRI signal, the residualised timeseries were temporally prewhitened using AR(p) model fitted to GLM residuals using Yule-Walker estimation [38]. A single global model order was selected empirically by session-wise comparison of candidate orders (*p* = 1–16) using Ljung-Box whiteness tests (lag 24). The smallest order meeting a pre-specified conservative QC target (≥ 90% of sessions with ≥ 95% parcels passing) was selected, yielding AR(4) (see Supplementary Fig. 2). Subsequently, session-wise task-related regression coefficients were estimated within a single spatial Bayesian GLM utilising R-INLA [39], where contrary to the reference pipeline [38], spatial dependence of parcel-wise effects was modelled using a Laplacian precision matrix derived from subject-specific structural connectome rather than a purely distance-driven mesh smoothness prior. This extension explicitly allowed for biologically informed long-range dependencies (e.g. cerebello-cortical coupling), directly addressing a limitation noted in the reference pipeline. All INLA fits converged without numerical failures, marginal likelihood was finite for all models (median −3.97 × 10^5^), and global fit metrics were stable (median Watanabe–Akaike information criterion (WAIC) 7.25 × 10^5^).

### Group-level Analysis

Task-related activation analysis was based on a second-level Bayesian hierarchical model implemented in Stan (see Supplementary Methods for full description). First-level INLA regression coefficients were collapsed into the main contrasts of interest: shooting–cross difference across all speed levels, linear speed scaling of the shooting–cross difference, and two analogous contrasts for the timing-error modulation. Contrast uncertainty was propagated from the full posterior covariance tensor of the first-level INLA coefficients to retain correlations between first-level regressors. The four main contrasts were modelled jointly within a single hierarchical model using clique-based regularisation – parcels were grouped into higher-order anatomical territories and modelled via clique pooling with parcel-level deviations (see Supplementary Table 1). The primary effects of interest for the four contrasts were: (i) control-group mean effect at the session-centred exposure level, and (ii) expert–control difference. The model further considered: (iii) linear session trend, (iv) group-by-session interaction, and (v) age and sex. Main effects of interest included parcel-specific deviation term, whereas nuisance terms were estimated at clique-level only (to improve model stability). Regression parameters were assigned weakly informative Gaussian priors; hierarchical scale parameters and the additional residual scale (variance beyond propagated first-level uncertainty) received half-normal priors. Posterior inference used Hamiltonian Monte Carlo (4 chains, 750 warm-up iterations and 750 sampling iterations per chain, target acceptance probability 0.90, maximum NUTS tree depth 12), yielding 3,000 posterior draws. Secondly, we performed a post-hoc evaluation of individual behavioural performance (average hit ratio in the shooting task) against the main contrast of interest (shooting vs cross) within controls only, after removing the second-level model effects, to evaluate whether functional patterns related to behavioural performance in controls match those distinguishing experts from controls.

Microstructural metrics (NDI, ODI and T₁ρ) were z-scored per parcel across subjects and modelled jointly within the same hierarchical clique-regularised structure as above (absent cortical parcels for T₁ρ handled via an observation mask), with equivalent sampling settings and priors. The main effect of interest was the expert-control group difference.

Both second-level Bayesian models exhibited good convergence and sampling behaviour ($\widehat{R}$ ≤ 1.01, high bulk/tail ESS, and no divergent transitions or tree-depth saturations; Supplementary Table 2).

Lastly, supplementary robustness analyses were performed to validate activation patterns detectable using the proposed Bayesian pipeline against previously published legacy methods [10] and to address potential concern of the hierarchical models overly favouring clique-level structure using an alternative model with explicitly prioritised parcel-level effects (see Supplementary Methods and Supplementary Fig. 3).

## Results

Behavioural analysis corroborated previously published results [12]. Experts exhibited higher accuracy in the main shooting task (median hit ratio [10th–90th percentile] of 0.68 [0.52–0.77]) than controls (0.53 [0.44–0.68]), largely overlapping performance in the control cross task (experts 0.86 [0.70–0.90] and controls 0.81 [0.72–0.91]) (see Fig. 1C) and similar difficulty (speed) scaling characteristics (see Fig. 1D).

### Task-related Activation Analysis

**Fig. 2:**
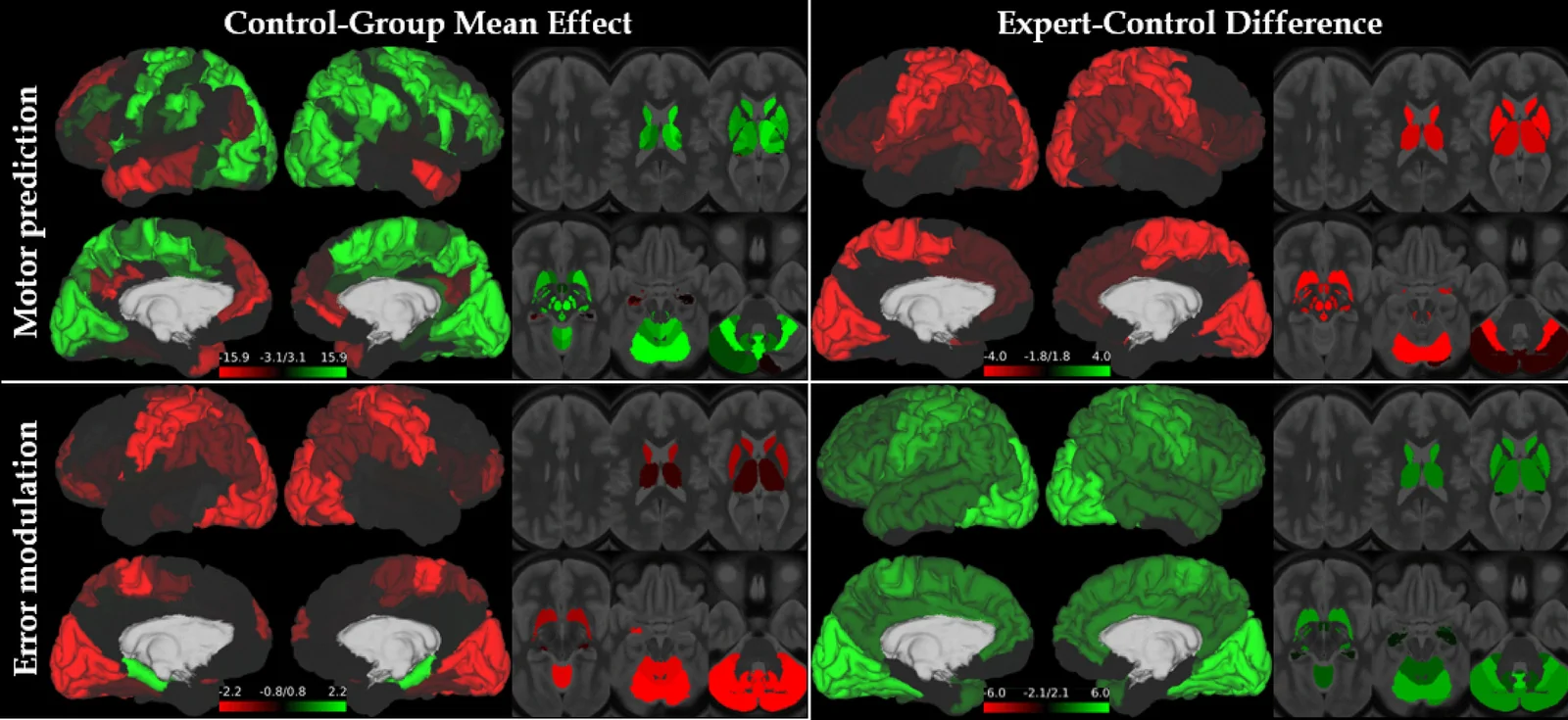
Results of second level task-related activation effects for motor prediction (shooting–cross) and error modulation (within the same contrast), separately for control-group mean effect and expert–control difference. Depicted as posterior medians (multiplied by 10^3^ to improve colour scale legibility) thresholded at a posterior sign probability ≥ 0.95. Motor prediction shows positive effects within sensorimotor, dorsal attention, visual, and frontoparietal control networks with coincident negative components in default-mode and episodic memory–related systems; error modulation in controls exhibits negative effects within the task-positive networks. Expert-control differences are dominated by relatively lower motor-prediction effect magnitude in experts, but higher activation associated with error magnitude. Shown in combined cerebral surface reconstructions with subcortical standard space in 6 slices (z = 16, 2, −12, −26, −40, −54). Laterality follows radiological convention (the right side of the image corresponds to the right side of the brain). See Table 1 for more detailed statistical results and anatomical localisation. Full numerical results underlying the figures are provided in the Supplementary Dataset

In Controls, **motor prediction** (shooting > cross) was associated with robust positive effects across the sensorimotor network, dorsal attention network, visual regions and frontoparietal control networks, encompassing also their subcortical and cerebellar elements (see Fig. 2, Table 1). Systematic negative components were detected within mostly midline default-mode network hubs and episodic memory related systems, such as hippocampus.

**Table 1:** Results of second level task-related activation effects for motor prediction (shooting–cross) and error modulation (within the same contrast). Corresponding spatial maps provided in the Fig. 2. Reported over larger anatomical partitions (cliques, see also Supplementary Table 1) separately for control-group mean effect and expert–control difference. The final column reports post-hoc analysis of the association of performance (average hit ratio during shooting task) and main motor-prediction contrast within controls evaluating whether functional patterns related to behavioural performance in controls match those distinguishing experts from controls. Values provided as posterior medians with 95% credible intervals (CrI) (multiplied by 10^3^ for numerical clarity) and posterior probability that the effect has the reported sign (P(sign)). Arrows indicate effects whose 95% CrI excludes zero (↑ positive, ↓ negative); effects with CrI spanning zero shown in grey. The ordering of cliques follows anatomical hierarchies across cortical, subcortical and cerebellar systems. Full numerical parcellated results are provided in the Supplementary Dataset

	Anatomical partition segment (clique)	Control-group mean effect						Expert-control difference						Performance association post-hoc** (controls)**		
		**Motor Prediction**			**Error modulation**			**Motor Prediction**			**Error modulation**					
			med [95% CrI]	P(sign)		med [95% CrI]	P(sign)		med [95% CrI]	P(sign)		med [95% CrI]	P(sign)		med [95% CrI]	P(sign)
Visual/Occipit. Cortex	**Primary Visual Cortex**	**↑**	16.9 [6.4,27.8]	0.999	↓	−4.0 [−6.6,−1.3]	0.998	↓	−5.7 [−10.2,−1.1]	0.990	↑	7.7 [4.4,11.0]	1.000	↑	7.0 [1.4, 12.1]	0.994
	MT + Complex and Neighbouring Visual Areas	**↑**	19.6 [15.9,23.0]	1.000	↓	−3.8 [−4.5,−3.0]	1.000	↓	−2.3 [−3.7,−0.8]	0.999	↑	6.2 [5.1,7.2]	1.000		3.8 [−1.2, 8.9]	0.927
	**Dorsal Stream Visual Cortex**	**↑**	23.8 [18.9,28.6]	1.000	↓	−2.3 [−3.3,−1.4]	1.000	↓	−3.9 [−5.7,−2.0]	1.000	↑	6.1 [4.8,7.4]	1.000	↑	5.7 [0.6, 11.3]	0.986
	Early Visual Cortex	**↑**	9.4 [3.3,15.7]	0.999	↓	−3.2 [−4.5,−1.9]	1.000	↓	−4.2 [−6.7,−1.6]	1.000	↑	6.5 [4.9,8.2]	1.000	↑	5.5 [0.6, 10.6]	0.984
	**Ventral Stream Visual Cortex**		3.2 [−1.0,7.4]	0.928	↓	−1.1 [−2.0,−0.3]	0.993		−1.6 [−3.3,0.1]	0.962	↑	5.6 [4.4,6.7]	1.000		1.8 [−3.0, 6.6]	0.770
Sensori-motor cort	Somatosensory and Motor Cortex	**↑**	6.2 [1.2,11.2]	0.994	↓	−2.2 [−3.2,−1.1]	1.000	↓	−4.4 [−6.5,−2.4]	1.000	↑	4.6 [3.3,6.0]	1.000		0.8 [−2.5, 4.0]	0.677
	**Premotor Cortex**	**↑**	19.0 [15.1,23.1]	1.000	↓	−2.1 [−3.0,−1.3]	1.000	↓	−5.4 [−7.1,−3.6]	1.000	↑	4.6 [3.4,5.7]	1.000		3.0 [−1.2, 7.5]	0.918
	Posterior Operculum		−1.0 [−5.6,3.3]	0.679		−0.5 [−1.4,0.4]	0.837	↓	−2.9 [−4.7,−1.2]	1.000	↑	3.4 [2.2,4.6]	1.000		0.0 [−3.9, 3.6]	0.501
	**Paracentral Lobular and Mid Cingulate**	**↑**	14.1 [10.1,18.1]	1.000	↓	−1.2 [−2.1,−0.4]	0.998	↓	−4.1 [−5.7,−2.5]	1.000	↑	3.8 [2.7,4.9]	1.000		1.5 [−2.5, 5.2]	0.754
Auditory Cortex	Early Auditory Cortex		1.9 [−3.1,6.9]	0.780		−0.1 [−1.1,0.9]	0.569	↓	−4.1 [−6.0,−2.1]	1.000	↑	2.4 [1.0,3.7]	1.000		0.8 [−2.8, 4.5]	0.677
	**Auditory Association Cortex**	**↓**	−5.3 [−9.4,−1.4]	0.997		−0.3 [−1.1,0.5]	0.784	↓	−2.5 [−4.0,−0.9]	0.998	↑	2.9 [1.8,3.9]	1.000		0.2 [−3.2, 3.5]	0.538
	Frontal Opercular and Insular Cortex	**↑**	6.5 [3.5,9.3]	1.000		−0.5 [−1.1,0.2]	0.920	↓	−3.6 [−4.8,−2.4]	1.000	↑	3.0 [2.2,3.9]	1.000		0.9 [−2.4, 4.2]	0.704
Tem. Cort	**Medial Temporal**		−1.0 [−6.1,3.7]	0.669	↑	3.0 [1.8,4.0]	1.000		−1.0 [−2.9,1.0]	0.841	↑	1.4 [0.1,2.8]	0.983		−0.8 [−5.0, 3.5]	0.643
	Lateral Temporal Cortex		−4.2 [−8.6,0.1]	0.972		−0.9 [−1.9,0.0]	0.972		−0.6 [−2.4,1.1]	0.739	↑	2.9 [1.7,4.2]	1.000		−0.8 [−4.4, 3.0]	0.681
Parietal Cortex	**Temporal-Parietal-Occipital Junction**	**↑**	8.9 [4.4,14.0]	1.000		0.3 [−0.8,1.3]	0.698	↓	−3.0 [−5.0,−1.2]	0.998	↑	3.1 [1.8,4.5]	1.000		1.8 [−2.8, 6.0]	0.796
	Superior Parietal and IPS	**↑**	23.9 [20.4,27.4]	1.000	↓	−1.4 [−2.1,−0.6]	1.000	↓	−4.9 [−6.3,−3.4]	1.000	↑	3.7 [2.7,4.7]	1.000	↑	4.2 [−0.8, 9.4]	0.951
	**Inferior Parietal Cortex**	**↑**	12.2 [8.5,15.6]	1.000	↓	−1.3 [−2.1,−0.5]	0.999	↓	−2.7 [−4.1,−1.3]	1.000	↑	3.5 [2.5,4.6]	1.000		1.4 [−2.8, 5.7]	0.742
	Posterior Cingulate		1.9 [−1.1,5.0]	0.890	↑	0.8 [0.2,1.4]	0.992		−1.1 [−2.3,0.1]	0.966	↑	3.0 [2.2,3.9]	1.000		1.2 [−2.5, 5.0]	0.744
Frontal Cortex	**Anterior Cingulate**		−0.1 [−3.1,2.8]	0.520		−0.4 [−1.1,0.3]	0.864	↓	−2.3 [−3.6,−1.1]	1.000	↑	3.5 [2.6,4.3]	1.000		0.5 [−2.6, 3.8]	0.625
	Orbital and Polar Frontal Cortex		−1.4 [−5.3,2.5]	0.760	↓	−1.5 [−2.4,−0.7]	0.999	↓	−2.3 [−4.0,−0.7]	0.998	↑	3.5 [2.4,4.6]	1.000		0.1 [−3.5, 3.4]	0.525
	**Inferior Frontal Cortex**	**↑**	5.4 [1.5,9.3]	0.999	↓	−0.9 [−1.8,−0.1]	0.989	↓	−2.2 [−3.8,−0.7]	0.997	↑	3.4 [2.4,4.4]	1.000		1.2 [−2.8, 5.1]	0.717
	Dorsolateral Prefrontal Cortex	**↑**	3.6 [0.6,6.8]	0.989		−0.6 [−1.3,0.0]	0.969	↓	−1.8 [−3.1,−0.7]	0.998	↑	2.9 [2.0,3.7]	1.000		0.6 [−2.8, 3.9]	0.627
Dienceph	**Thalamus**	**↑**	11.0 [6.4,14.7]	1.000	↓	−1.1 [−2.0,−0.2]	0.989	↓	−3.6 [−5.3,−1.9]	1.000	↑	3.7 [2.6,4.8]	1.000		1.6 [−388.4, 353.7]	0.503
	Hypothalamus		7.5 [−0.5,15.2]	0.968		−0.3 [−1.9,1.3]	0.659	↓	−3.5 [−6.6,−0.4]	0.987	↑	2.3 [0.3,4.4]	0.990		−1.2 [−365.4, 397.8]	0.504
Basal Ganglia	**Striatum**	**↑**	12.3 [8.2,16.4]	1.000	↓	−1.6 [−2.5,−0.8]	1.000	↓	−4.4 [−6.1,−2.8]	1.000	↑	4.3 [3.2,5.3]	1.000		1.9 [−366.9, 369.9]	0.510
	Pallidum	**↑**	11.4 [3.6,19.0]	0.997		−1.1 [−2.7,0.5]	0.919	↓	−4.0 [−6.9,−0.9]	0.993	↑	3.5 [1.5,5.6]	0.999		−3.5 [−368.3, 366.4]	0.514
	**Basal Ganglia-Associated Nuclei**	**↑**	14.4 [6.3,21.7]	1.000		−0.8 [−2.5,0.9]	0.836	↓	−3.5 [−6.7,−0.5]	0.987		2.2 [0.0,4.5]	0.973		−2.0 [−390.5, 394.3]	0.507
	Brainstem – Reticular and Sensorimotor Nuclei	**↑**	14.0 [6.9,21.1]	1.000		0.9 [−0.8,2.6]	0.850	↓	−5.2 [−8.1,−2.3]	1.000		1.3 [−0.7,3.3]	0.880		1.5 [−376.2, 376.2]	0.507
Limb. Syst	**Amygdala**		−0.9 [−7.4,5.4]	0.608		0.4 [−1.1,1.9]	0.687	↓	−3.6 [−6.2,−0.9]	0.995	↑	2.4 [0.4,4.3]	0.990		−2.2 [−414.5, 389.4]	0.509
	Hippocampus		−4.4 [−9.2,0.7]	0.952		−0.6 [−1.7,0.4]	0.890		0.9 [−1.0,2.9]	0.830	↑	1.9 [0.6,3.3]	0.997		0.4 [−365.6, 386.0]	0.501
Cerebellum	**Cerebellum – Anterior Sensorimotor**	**↑**	11.3 [3.4,19.4]	0.998	↓	−2.0 [−3.6,−0.4]	0.995		−2.7 [−5.7,0.4]	0.954	↑	3.1 [1.2,5.1]	0.999		2.5 [−374.4, 419.6]	0.510
	Cerebellum – VI (Transitional)	**↑**	19.2 [10.7,28.3]	1.000	↓	−2.5 [−4.7,−0.2]	0.983	↓	−4.8 [−9.0,−0.6]	0.989	↑	4.6 [2.0,7.4]	1.000		4.1 [−378.3, 362.5]	0.517
	**Cerebellum – Executive/Associative**	**↑**	11.5 [6.4,17.1]	1.000	↓	−3.6 [−4.7,−2.5]	1.000	↓	−2.2 [−4.4,−0.2]	0.983	↑	4.0 [2.6,5.5]	1.000		2.0 [−391.7, 380.4]	0.508
	Cerebellum – Sensorimotor VIII	**↑**	13.9 [7.6,20.4]	1.000	↓	−4.1 [−5.4,−2.7]	1.000		−1.6 [−4.2,1.0]	0.878	↑	5.3 [3.7,6.9]	1.000		4.2 [−372.6, 408.2]	0.521
	**Cerebellum – Posterior (IX–X)**	**↑**	10.9 [4.5,17.2]	1.000	↓	−2.3 [−3.6,−1.0]	1.000		−1.2 [−3.8,1.3]	0.827	↑	2.9 [1.2,4.6]	1.000		−1.8 [−408.9, 389.6]	0.510
	Cerebellar Deep Nuclei		2.4 [−5.1,9.8]	0.746	↓	−2.0 [−3.6,−0.5]	0.994		−0.4 [−3.5,2.9]	0.600		1.8 [−0.3,3.7]	0.953		−1.1 [−395.3, 364.1]	0.508

By contrast**, error modulation** in the control cohort was characterised by predominantly negative effects within the same sensorimotor, attention- and control-related networks shown to be positively engaged by the motor prediction contrast. Furthermore, regions negatively modulated during motor prediction, such as mesiotemporal cortex and posterior cingulate, exhibited a reversal of their prediction-associated suppression.

**Group comparison** over the motor prediction contrast indicated narrower dynamic range between task-positive and task-negative components in experts, as evidenced by their attenuated responses within main cortico-subcortical sensorimotor and dorsal parietal networks. Moreover, the post-hoc association between hit ratio and motor prediction contrast in controls was relatively weak and generally positive (Table 1), thus arguing against the expertise effect being a simple derivative of better behavioural performance in the group. On the other hand, experts showed relatively higher response magnitude to increasing deviation from the task optimum within large-scale frontoparietal and visuospatial networks as compared with controls.

**Fig. 3:**
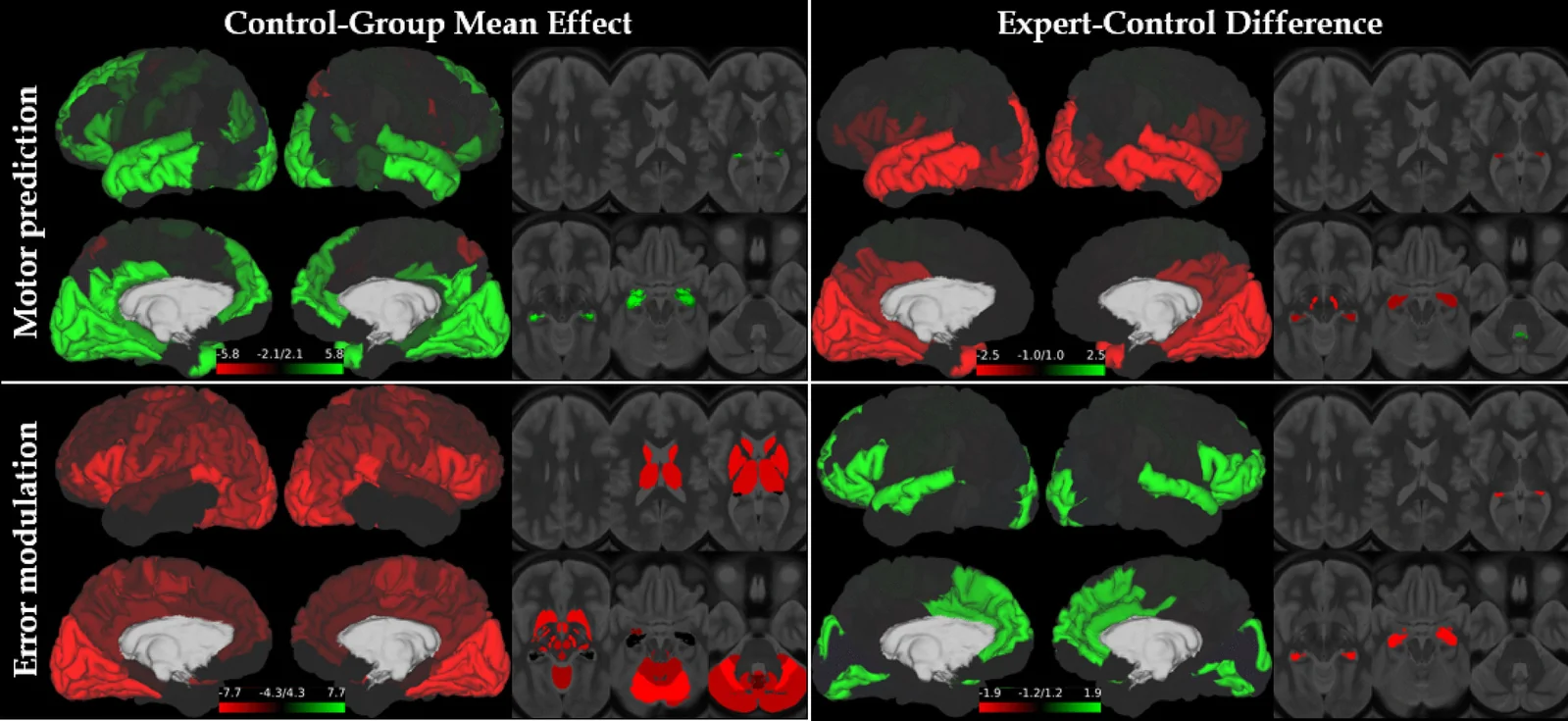
Results of second level task-related activation effects for difficulty (speed) scaling of motor prediction (shooting–cross) and error modulation (within the same contrast), separately for control-group mean effect and expert–control difference. Depicted as posterior medians (multiplied by 10^3^ to improve colour scale legibility) thresholded at a posterior sign probability ≥ 0.95. Speed scaling of motor prediction is expressed mostly within frontotemporal, visuospatial networks and limbic structures, while in error-modulation, it shows largely negative effect in controls. Experts exhibited relatively lower extent of speed-dependent modulation of predictive timing in temporal and visual cortices, but relatively higher error-related responses with increasing speed mostly frontally. Shown in combined cerebral surface reconstructions with subcortical standard space in 6 slices (z = 16, 2, −12, −26, −40, −54). Laterality follows radiological convention (the right side of the image corresponds to the right side of the brain). See Supplementary Table 2 for more detailed statistical results and anatomical localisation. Full numerical results underlying the figures are provided in the Supplementary Dataset

**Difficulty (speed) scaling** analysis revealed predominantly positive control-group mean effect over frontotemporal and visuospatial networks, with additional involvement of limbic system components (see Fig. 3, Supplementary Table 3). Other subcortical structures showed posterior credible intervals largely centred around zero. Response to error with rising speed was negative, with spatial distribution similar to the speed-independent error-modulation effect. When compared to controls, experts exhibited attenuated speed-dependent motor prediction response, mostly in the occipito-temporal cortices, but also in hippocampal region. In contrast, speed scaling of error modulation was higher in experts in the mesiofrontal and secondary visual cortex.

### Microstructural Group Comparison

Experts showed modestly, but spatially consistent higher NDI and ODI relative to controls within dorsal attention, sensorimotor, and frontoparietal control networks, while differences in NDI over midline default-mode network hubs and frontal cortico-subcortical loops were detected only in NDI. On the other hand, cerebellum exhibited a mixed microstructural profile, with higher NDI in experts dominantly in lobules VI and VIII, co-occurring with oppositely directed ODI and T₁ρ effects, mostly over cerebellar crura and lobule VIII. Lastly, limbic system showed slower T₁ρ relaxation and lower ODI in experts, whereas NDI credible intervals were effectively zero-centred.

## Discussion

The presented results indicate that intensive motor training is associated with a reconfiguration of large-scale networks – both their dynamical patterns and microstructural foundations. Higher behavioural performance in experts was associated with reduced reliance on primary task-positive sensorimotor and visuospatial network activation and enhanced error-related response within overlapping structures. This framework aligns well with the established principle of cerebral networks becoming more efficient at generating accurate neural outputs with practice [40], where available processing capacity is hypothesised to be redistributed towards deviation management and integrative control.

### Conceptual Shift from Cerebellar Dominance to Distributed Predictive Control Networks

In our earlier studies employing the same predictive timing paradigm [10, 11, 41, 42], cerebellum emerged as the dominant neural correlate of motor prediction, supporting its role as the principal driver within short-scale predictive timing processes. The presented structural connectome-informed hierarchical analysis reveals a more distributed architecture encompassing large-scale cortico-subcortical systems. Even general population without long-term motor training (see Fig. 2 – Control-group mean effect) processes temporal prediction over sensorimotor, visuospatial, attentional and frontoparietal control systems together with striatal, thalamic and cerebellar loops, while disengaging default-mode network hubs. Predictive control thus emerges as an inherently network-based algorithm. Rather than constituting a singular dominant region, the cerebellum thus seems to occupy a position deeply embedded within a larger hierarchical architecture. This concept is well aligned with the emerging view of motor learning, process optimisation and general cerebral processing patterns as an interplay between distributed networks with hierarchical organisation and information transfer, rather than the function of a single dominant node [4, 43]. Interestingly, the quantitative deviation from the task optimum was associated with down-modulation of this predictive network, possibly indicating weaker or less stable engagement of internal models with larger prediction errors rather than the recruitment of error-driven corrective processes described previously [6]. Importantly, the distributed cortical networks exhibiting error-related modulation may encompass the representation and formation of error signals themselves rather than the hypothesised corrective process. Nonetheless, the spatial and temporal resolution of the available data is unable to distinguish such subprocesses within the presented aggregate associations.

### Expertise-associated Differences in Predictive and Error-related Dynamics

One of the main findings of this study – reduction in prediction-related functional signal with expertise combined with superior behavioural performance – is consistent with more efficient neural dynamics within an established architecture rather than the emergence of qualitatively distinct networks. These findings thus extend previous reports of decreased functional activity in highly skilled musicians, generally limited to motor areas in simple motor tasks [44–46], to reverberations across large-scale networks encompassing dorsal parietal and cortico-subcortical sensorimotor loops (see Fig. 2 – Expert–Control Difference).

This pattern was further supported by the difficulty-dependent effects (see Fig. 3). Regions exhibiting relative down-regulation in the main task in controls, including temporal, limbic and prefrontal systems, were progressively engaged with rising task demands, possibly indicating adaptive dynamic recruitment of higher-order supervisory systems when feedforward prediction models were becoming insufficient. Importantly, this scaling effect was markedly attenuated in experts who showed stronger engagement of these regions with increasing error magnitude.

However, our initial hypothesis of error signal minimisation with expertise-driven optimisation of internal prediction models and hence their presumed higher accuracy was not confirmed. Contrary to that, the observed patterns may actually reflect redistribution of processing capacity from dominantly predictive, feedforward regime towards the management of departures from the expected optimum [47]. Neurobiologically, this interpretation is compatible with efficient coding concepts, where brain reduces redundancy in neural signals within established pathways and reallocates processing resources to unexpected information [48].

Given the distributed characteristics of both expertise-related effects, predictive timing program optimisation does not seem to be dominated by one process or one anatomical region, resonating with the concept of super-learning [4, 49]. In contrast to more fragmented notions attributing cerebellar architecture to supervised learning [50], basal ganglia to reinforcement learning [51] and cortex to unsupervised learning [52], this view proposes the brain as a synergic, hierarchical integrative system being iteratively updated through feedback mechanisms and predictive error-based correction.

### Expertise-related Microstructural Characteristics of Predictive Networks

**Fig. 4:**
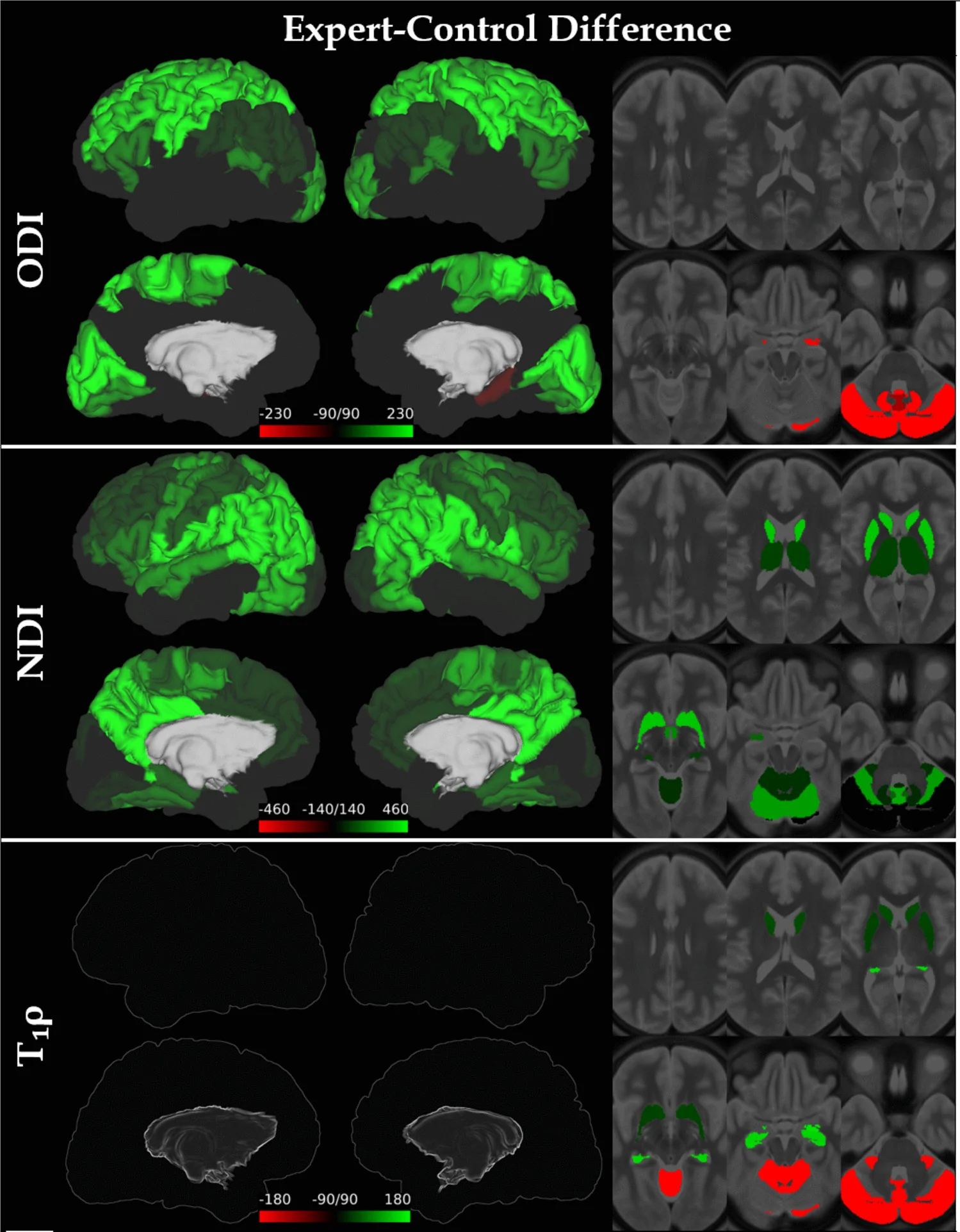
Inter-group comparison of microstructural metrics – Neurite Density Index (NDI), Orientation Dispersion Index (ODI) and adiabatic T₁ρ relaxation constant (analysed only in subcortical structures due to the field of view utilised in the MRI acquisition). Depicted as posterior medians (multiplied by 10^3^ to improve colour scale legibility) thresholded at a posterior sign probability ≥ 0.95. Experts showed relatively higher ODI and NDI over visual, dorsal attention, sensorimotor, and frontoparietal control networks, but exhibited a relatively mixed microstructural profile in the cerebellum. Shown in combined cerebral surface reconstructions with subcortical standard space in 6 slices (z = 16, 2, −12, −26, −40, −54). Laterality follows radiological convention (the right side of the image corresponds to the right side of the brain). See Supplementary Table 3 for more detailed statistical results and anatomical localisation. Full numerical results underlying the figures are provided in the Supplementary Dataset

The microstructural profile associated with expertise revealed spatially coherent, but only modestly higher NDI and ODI over sensorimotor, frontoparietal control and dorsal attention networks (see Fig. 4), in line with growing evidence of cortical microstructural remodelling accompanying skill acquisition [53, 54]. Although NODDI is inherently ill-posed for fully resolving microstructural complexity of grey matter at the spatial resolution of available DWI protocols, converging evidence from various acquisition schemes, analytical approaches and biological models indicates dendritic architecture as a dominant contributor of cortical diffusion indices [55, 56]. While highly inferential in nature, higher NDI and ODI in experts may be seen as markers of increased dendritic arborisation complexity, reflecting experience-related reconfiguration as a putative backbone of optimised predictive networks. Indeed, learning-related plasticity has been shown to include selective pruning of dendritic spines and connection strengthening [57, 58].

Compared to this relatively uniform cortical pattern, the cerebellum exhibited substantial divergence in individual metrics – relatively higher NDI with lower ODI and T₁ρ, particularly within the cerebellar crura. This dissociation likely stems from its highly specialised architecture and processing patterns uniquely suited for algorithmic corrections and reconciliation of predicted sensorimotor consequences of planned actions with observed outcomes [9]. Massive parallelisation, highly regular microcircuitry, dense parallel fibre organisation and climbing fibre-driven synaptic plasticity not only allow for rapid update of internal models [59, 60], but may predispose the cerebellum to embrace selective increase of directional coherence within dominant fibre populations as a substrate of program fine-tuning. Concomitantly shorter T₁ρ relaxation time points to enhanced interactions between water protons and the macromolecular environment [61–63], compatible with the concept of experience-dependent synaptic remodelling and intracellular processes related with increased plasticity [64].

### Limitations and Methodological Considerations

While the presented analytical framework enabled the detection of distributed effects of network optimisation that would not be accessible using conventional voxel-wise mass-univariate approaches, the cost reflected in computational and methodological complexity is substantial. High-resolution surface-based analysis using the HCP pipeline combined with probabilistic structural connectome reconstruction required extensive subject-level preprocessing, while hierarchical Bayesian model (see Supplementary Methods), integrating network-regularised spatial inference with fully generative multi-level modelling of interacting task components, extends beyond the scope of commonly employed neuroimaging packages.

This level of complexity represents both the major strength and the primary limitation of the presented study. Advanced parcellation clearly improved cortical effect localisation and helped preserve network organisation compared to standard-space atlas pipelines, although acquisition characteristics likely contributed substantially since even simpler voxel-wise legacy analysis revealed a more distributed network pattern than reported in previous studies (see Supplementary Fig. 3). Ultimately, subtle, but coherent distributed effects became stable only under a unified generative model with proper propagation of uncertainty over analysis levels and across parcels, with inference constraints along biologically meaningful pathways derived from structural connectome replacing the standard, purely local spatial dependence assumptions. Nonetheless, statistical modelling cannot fully disentangle all processes intertwined within experimental paradigms, including carry-over and feedback effects from previous trials, subjective relevance of positive and negative outcome feedback, salience or attentional responses and possible inter-individual differences in employed task strategies. Consequently, presented contrast effects should be understood as aggregate associations rather than the signature of specific computational programmes.

Additionally, the cross-sectional design comparing experts and controls precludes causal inference about expertise-driven network reorganisation since pre-existing neurobiological predispositions may contribute non-negligibly both to microstructural and functional profiles. However, contrary to the conditions in North America, athletic selection pressure is likely substantially lower in the present regional context where baseball is not a dominant sport. Secondly, the generalisability of detected optimisation patterns to other predictive and fine-tuning processes remains to be fully established, although previous unimodal studies in different populations have reported isolated components consistent with present findings – both expertise-related attenuation of functional activity [44–46] and ODI increase [53]. Finally, all MRI metrics are conceptually only indirect, model-dependent proxies of underlying biology. The BOLD signal reflects haemodynamic processes rather than true neural activity and may therefore be affected by group differences in vascular dynamics – an important consideration for the comparison of athletes and non-athletic controls. However, superior vascular fitness would be expected to produce higher rather than attenuated BOLD response in experts in the main motor prediction contrast, strengthening the proposed interpretation of more efficient network optimisation.

## Conclusion

In summary, this multimodal synthesis shows that expertise is associated with distinct functional and microstructural characteristics, implicating optimisation of predictive mechanisms at network-wide levels. The combination of lower task engagement with higher error sensitivity may indicate expertise-driven shift from dominantly predictive to error-corrective mode. Furthermore, its combination with microstructural alterations is consistent with experience-driven plasticity supporting these dynamics. Future longitudinal studies investigating the joint development of functional optimisation, its underlying microstructural changes, as well as their coupling, will be essential to elucidate this concept.

## Supplementary Information

Below is the link to the electronic supplementary material.

Supplementary file1 (ZIP 37766 KB)

Supplementary file2 (PDF 1.05 MB)

## Data Availability

Fully pre-processed parcellated MRI output (timeseries, NDI, ODI, T₁ρ), demographical information, behavioural performance, analytic outputs and supporting data values for presented figures are available as Supplementary Dataset. De-identified raw MRI data will be provided based on qualified academic request to the corresponding author.

## Abbreviations

AP: Anterior–posterior
AR: Autoregressive (model)
ARAS: Ascending reticular activating system
ATAG: Atlas of the basal ganglia
BOLD: Blood-oxygen-level-dependent
CIFTI: Connectivity Informatics Technology Initiative
DWI: Diffusion-weighted imaging
DVARS: Temporal derivative of timecourses root mean square variance
ESS: Effective sample size
FD_95_: 95Th percentile of framewise displacement
fMRI: Functional magnetic resonance imaging
FA: Flip angle
GLM: General linear model
GM: Grey matter
GRAPPA: Generalized Autocalibrating Partial Parallel Acquisition
HCP: Human Connectome Project
HRF: Haemodynamic response function
ICA: Independent component analysis
INLA: Integrated nested Laplace approximations
iFOD2: Second-order integration over fibre orientation distributions
MB: Multiband
MNI: Montreal Neurological Institute
MPRAGE: Magnetization Prepared Rapid Acquisition Gradient Echoes
MRI: Magnetic resonance imaging
NDI: Neurite density index
NODDI: Neurite orientation dispersion and density imaging
NUTS: No-U-Turn Sampler
ODF: Orientation distribution function
ODI: Orientation dispersion index
PA: Posterior–anterior
PALM: Permutation analysis of linear models
QC: Quality control
$\widehat{R}$: Gelman–Rubin convergence statistic
SIFT2: Spherical-deconvolution informed filtering of tractograms 2
SPACE: Sampling Perfection with Application-optimised Contrasts using different flip angle Evolutions
SPM: Statistical Parametric Mapping
TE: Echo time
TI: Inversion time
TR: Repetition time
tSNR: Temporal signal-to-noise ratio
T₁ρ: Adiabatic longitudinal relaxation in the rotating frame
WAIC: Watanabe–Akaike information criterion
