# Supplementary material for: Motor Expertise Combines Lower Task Engagement with Enhanced Error Sensitivity
Source: Cerebellum. 2026 Nov 4;25(6):167. doi: 10.1007/s12311-026-02100-4 (PMC13736444; doi:10.1007/s12311-026-02100-4)
Supplement: Supplementary file 1 [file 12311_2026_2100_MOESM2_ESM.pdf]

# Supplementary Methods

## Second-level hierarchical model (full formula)

### Indices and data

Let

- $m = 1, \dots, M$ index participants ($M$ = 87),
- $s = 1, \dots, S$ index sessions ($S$ = 3),
- $p = 1, \dots, P$ index parcels ($P$ = 432),
- $k = 1, \dots, K$ index planned contrasts ($K$ = 4)
  - shooting–cross difference across all speed levels,
  - linear speed scaling of the shooting–cross difference,
  - shooting–cross difference in timing-error modulation,
  - linear speed scaling of shooting–cross difference in timing-error modulation
- $c(p) \in \{1, \dots, C\}$ anatomical clique of parcel $p$ ($C$ = 37, see Supplementary Table 1).

INLA inputs to the second level group model are the session- and parcel-wise contrast estimates and their propagated uncertainties:

- $\hat{\beta}_{mspk}$ = first-level (INLA) posterior mean of contrast $k$ for participant $m$, session $s$, parcel $p$.
- $\mathrm{SE}_{mspk}$= propagated posterior standard deviation for that contrast based on the full posterior covariance tensor of first-level coefficients.

Participant/session predictors:

- $G_m \in \{expert, control\}$: group indicator
- $A_m$: age (centred and scaled)
- $X_m \in \{male, female\}$: sex indicator
- $t_s \in \{-1, 0, 1\}$: centred session code (linear practice trend)

The second level was a multivariate model over all contrasts: $k$ = 1, …, $K$ were estimated jointly in one model with contrast-specific parameters $(\cdot)_k$ and shared hierarchical structure across parcels/cliques, but with contrast-specific parameters and conditionally independent likelihood across contrasts.

### Observation model (measurement-error likelihood)

For every $m, s, p, k$:

$$\hat{\beta}_{mspk} \sim \mathcal{N}\left(\mu_{mspk}, \mathrm{SE}^2_{mspk} + \sigma^2_{\mathrm{add},k}\right),$$

where $\sigma_{\mathrm{add},k} \geq 0$ reflects extra second-level variability over the propagated first-level uncertainty.

### Linear predictor with clique pooling

Clique-level coefficient matrices were defined for each contrast $k$ as follows:

- control-group mean effect at $t$ = 0: $\alpha_{0,ck}$ (parameterised at the exposure level centred to the middle session ($t$ = 0) to improve interpretability; all parameters were estimated jointly using data from all three sessions).
- expert–control difference at $t$ = 0: $\alpha_{G,ck}$

- linear session trend in controls: $\alpha_{S,ck}$
- group-by-session interaction: $\alpha_{GS,ck}$
- age effect (clique-only): $\alpha_{A,ck}$
- sex effect (clique-only): $\alpha_{X,ck}$

Furthermore, parcel-level deviation was added for the following terms:

- unstructured deviations for control-group mean effect and expert-control difference: $\varepsilon_{pk}^{(0)}$, $\varepsilon_{pk}^{(G)}$
- deviations for session terms: $\delta_{pk}^{(S)}$, $\delta_{pk}^{(GS)}$

with hierarchical scales controlling the extent of parcel-level deviations around clique-level pooled effects (contrast-specific, non-negative; smaller values imply stronger pooling): $\sigma_{\varepsilon 0,k}$, $\sigma_{\varepsilon G,k}$, $\sigma_{\delta S,k}$, $\sigma_{\delta GS,k}$.

Then the mean for each observation, estimating all planned contrasts $k = 1, \ldots, K$ under the same hierarchical structure, was:

$$\mu_{mspk} = \underbrace{\left(\alpha_{0,ck} + \sigma_{\varepsilon 0,k}\, \varepsilon_{pk}^{(0)}\right)}_{\text{control-group mean effect}} + \underbrace{\left(\alpha_{G,ck} + \sigma_{\varepsilon G,k}\, \varepsilon_{pk}^{(G)}\right) G_m}_{\text{expert-control difference}} + \underbrace{\left(a_{S,ck} + \sigma_{S,k}\, z_{pk}^{(S)}\right) t_s}_{\text{linear session trend}} + \underbrace{\left(a_{GS,ck} + \sigma_{GS,k}\, z_{pk}^{(GS)}\right) (G_m t_s)}_{\text{group-by-session interaction}} + \underbrace{\alpha_{A,c(p)k} A_m + \alpha_{X,c(p)k} X_m}_{\text{age/sex adjustment}}$$

## Priors

Weakly informative priors were utilised, independently across contrasts *k:*

- For all cliques $c$ and contrasts $k$: $\alpha_{\cdot,ck} \sim \mathcal{N}(0,1)$ for $\cdot \in \{0, G, S, GS, A, X\}$.
- Parcel-level latent deviations: $\varepsilon_{pk}^{(0)}$, $\varepsilon_{pk}^{(G)}$, $\delta_{pk}^{(S)}$, $\delta_{pk}^{(GS)} \sim \mathcal{N}(0,1)$.
- Hierarchical scales and the additional residual (weakly informative scale considering the structural connectome-based spatial regularisation on parcel-wise effects in the first-level INLA model): $\sigma_{\varepsilon 0,k}$, $\sigma_{\varepsilon G,k}$, $\sigma_{\delta S,k}$, $\sigma_{\delta GS,k}$, $\sigma_{\text{add},k} \sim \mathcal{N}^{+}(0,0.2)$

## Robustness analysis of clique-pooling

To address potential concern of the hierarchical models overly favouring clique-level structure, an alternative model was fitted with explicitly prioritised parcel-level effects via an estimated clique-weight controlling the extent of clique-level pooling. Specifically, the parcel-level parameter was modelled as a convex combination of the clique component and an independent parcel component:

$$\mu_{\cdot p,k} = \kappa_{\cdot}\, a_{c(p),k} + (1 - \kappa_{\cdot})\, b_{\cdot p,k}$$

where $a_{c(p),k}$ stands for clique-level effects, $b_{p,k}$ parcel-level effects, and $\kappa \in [0,1]$ is an estimated weighting factor of clique influence. Furthermore, priors on $\kappa \sim \beta(1,9)$ strongly favoured weak clique contributions by default (mean 0.10), so that parcel-level resolution would collapse to clique-dominant outcome only if strongly supported by the data. The other parameters were retained from the primary model.

This robustness model produced spatial patterns and outcomes virtually identical to the primary hierarchical model, with very low posterior estimates of clique weights in the expert–control effect ($\kappa_G$ posterior mean ≈ 0.0026, $5^{th}$–$95^{th}$ percentile ≈ 0.0023–0.0029; $\hat{R} \approx 1.01$). Ergo, the model did not primarily rely on explicit clique pooling. However, parcel-specific deviations were still strongly shrunk towards zero and clique component explained the majority of the group-effect posterior mass (mean share of clique contribution ≈ 0.95; $5^{th}$ – $95^{th}$ percentile ≈ 0.884–0.996). While this apparent dominance of lower granularity of anatomical resolution may genuinely reflect a smooth biological effect, another explanation may lie in the insufficient sensitivity at parcel resolution (effective sample size, temporal signal-to-noise ratio) where available dataset cannot support inferences at higher resolution.

## Comparison against Legacy Pipelines

To validate activation patterns detectable using the proposed Bayesian pipeline, a legacy-style analysis was performed restricted to the cohort-level task effect (shooting–cross averaged across speed levels and across all three task fMRI sessions) corresponding to the previously published "Positive Effect of Task" (Filip et al. 2017). Three complementary analyses were performed:

- SPM-style mass univariate voxel-wise analysis in MNI space using HCP-pipeline volumetric output and common spatial smoothing approach (8mm isotropic Gaussian kernel), with first and second-level models estimated in SPM12 (Friston et al. 1994) using default settings as in our previous publication (Filip et al. 2017)
- Parcellated analysis, mass univariate analysis over 432 parcels passing QC described in the main text, first-level model in SPM12 (no global normalisation, AR(1), masking threshold 0.8, no interactions terms, canonical HRF convolution), second-level inference via PALM (10,000 permutations) (Winkler et al. 2014).
- simplified Bayesian model retaining structural connectome regularised first-level INLA estimation, uncertainty propagation and clique pooling, but limited at the second level to single legacy task-effect contrast.

The frequentist models implemented standard significance threshold of α=0.05 with family-wise error correction (voxel/parcel-level) for multiple comparisons due to the large number of independent tests (>$1.5\times10^5$ non-zero voxels within SPM-derived study-specific brain mask; 432 parcels). In contrast, joint estimation with partial pooling in Bayesian hierarchical models inherently shrinks extreme estimates towards group-level distribution, thereby mitigating multiplicity concerns (Gelman et al. 2012). Descriptive localisation for the Bayesian model was thresholded at 95% central credible interval – i.e. posterior sign probability of 97.5% ($\max[P(\theta > 0), P(\theta < 0)] \geq 0.975$), corresponding to the frequentist two-sided 5% convention (Gelman et al. 2013).

# Supplementary Figures

**Supplementary Figure 1: Parcels excluded based on multivariate parcel-level quality control procedure.** Excluded regions were located primarily in the orbitofrontal and polar frontal cortex, anterior cingulate cortex, lateral and medial temporal cortex (including entorhinal and perirhinal areas), brainstem, cerebellar lobule VIIIa, and the basolateral ventral amygdala. Subcortical structures presented in 6 slices z = 16, 2, -12, -26, -40, -54 (Montreal Neurological Institute space), consistent with subsequent figures. Laterality convention follows radiological standards (the right side of the image corresponds to the right side of the brain).

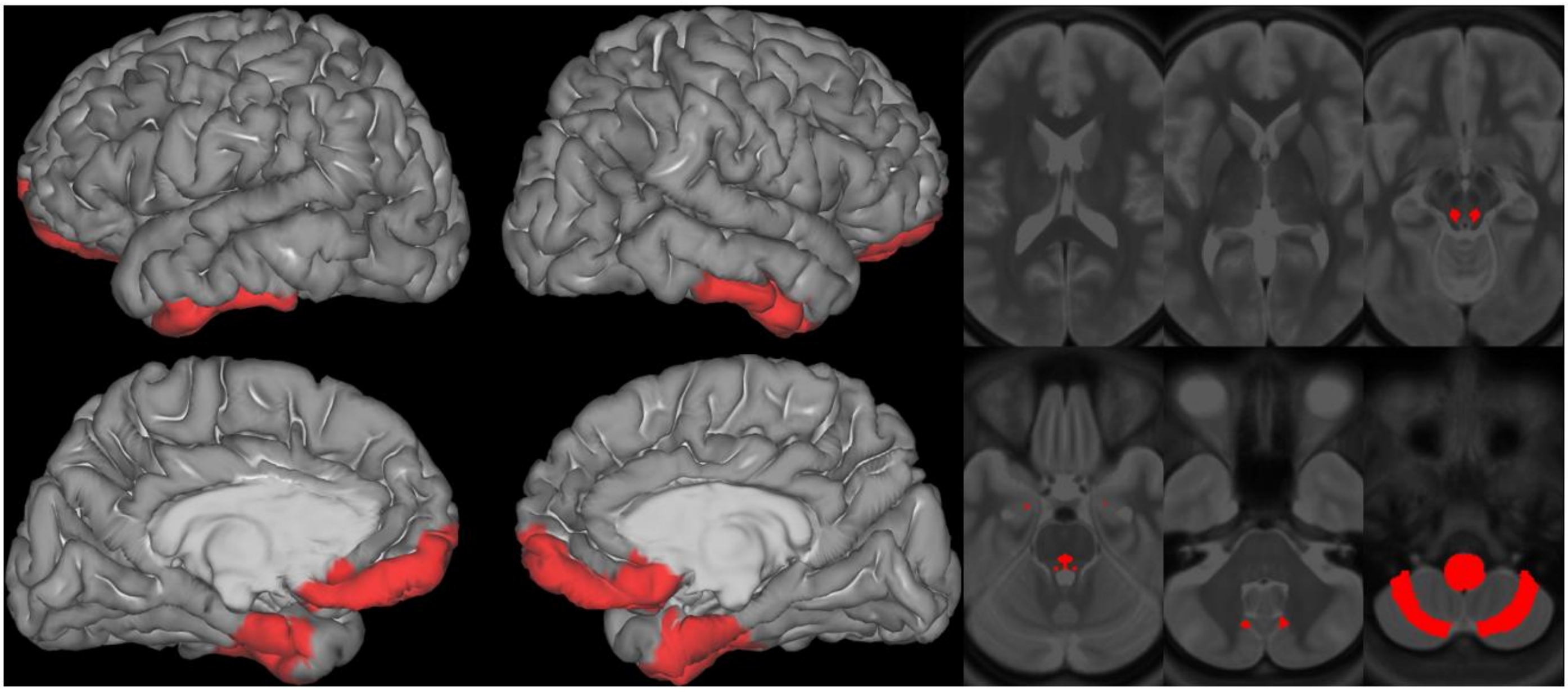

**Supplementary Figure 2: Selection of the global autoregression (AR) order for temporal prewhitening,** based on session-wise assessment of candidate AR orders (p) using Ljung–Box tests (lag 24), with Benjamini–Hochberg correction across parcels within each session (FDR-adjusted α = 0.05). The curve corresponds to the fraction of sessions with at least 95% of parcels meeting the whiteness criterion. The dashed horizontal line marks the prespecified target (≥0.90 of sessions); the dashed vertical line indicates the selected order (AR(4), i.e. the smallest p meeting the criterion).

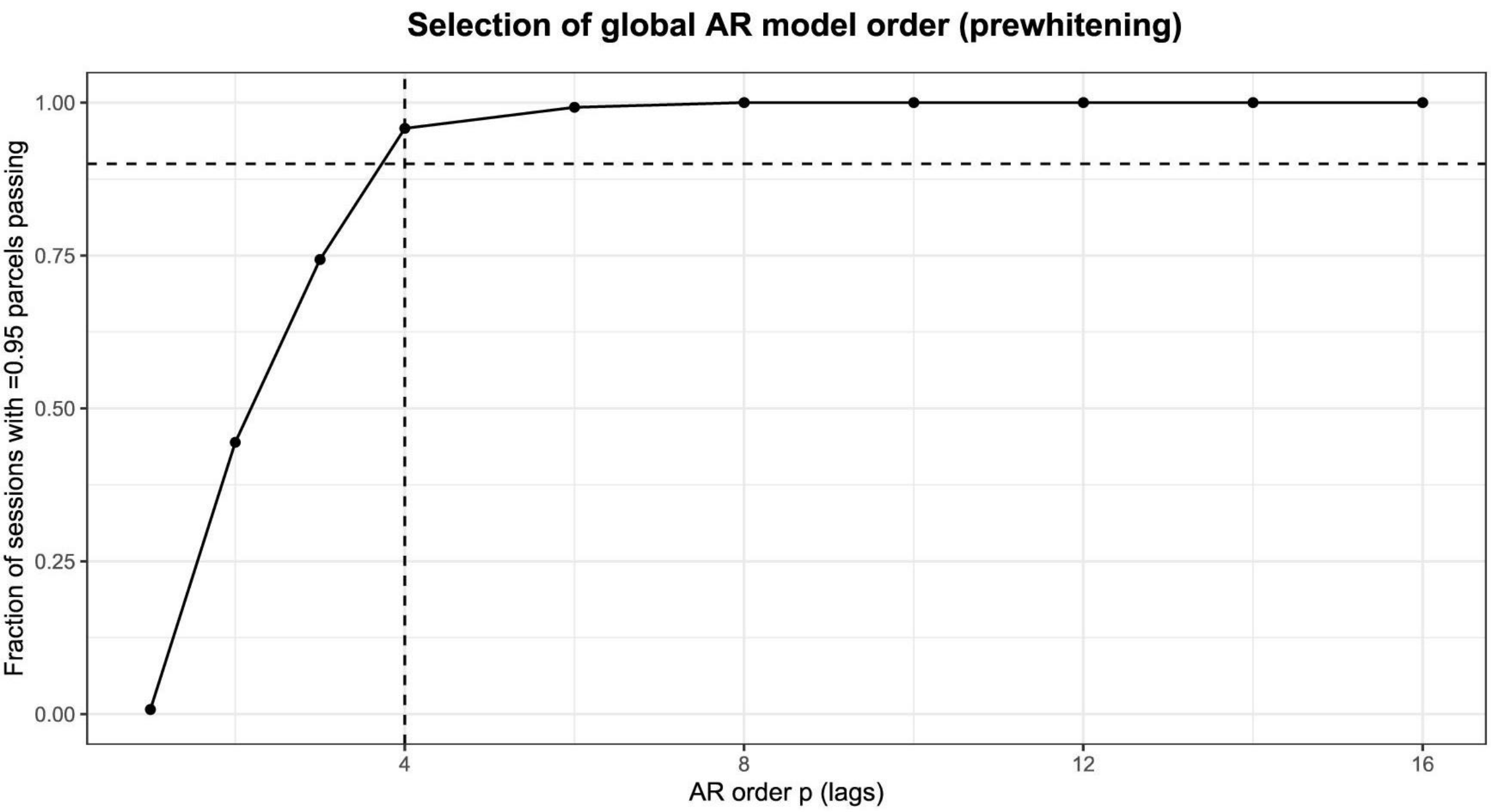

**Supplementary Figure 3: Comparison against legacy pipelines (voxel/parcel-wise frequentist approach).** Whole-cohort task-related contrast (shooting–cross averaged over speeds and sessions at each subject's first-level model). SPM12 (mass-univariate voxel-wise analysis in standard space) and mainly PALM (mass-univariate parcel-level analysis) provided highly consistent cortical and subcortical activation patterns as the Bayesian pipeline. Subcortical effects included increase in striatal, thalamic and cerebellar activity, including the replication of right crus II decrease in otherwise relatively symmetric settings. Cortical effects showed increased activity in premotor, superior temporal, visual and dorsolateral prefrontal cortex and decreases in cingulate, frontal polar and lateral temporal cortex. The close correspondence between PALM and Bayesian activation patterns reflects high robustness of the task-related contrast at the level of the whole cohort, driving mass-univariate inference towards near-ceiling significance. Simple thresholding then collapses calibrated effect estimates and uncertainty quantification provided by the Bayesian pipeline, which are shown to be essential in the primary analyses in the main text.

SPM12 and PALM maps displayed using the common convention overlaying two separate positive and negative directional contrasts thresholded at $p<0.05$ (one-sided; union of tails) for pattern comparison. Bayesian results thresholded using posterior sign probability ≥0.95 (sign-symmetric by construction; roughly analogous to two-sided $p\approx0.05$ threshold; though not mathematically equivalent to frequentist tail probability). SPM12 results presented in 8 axial slices (z = 44, 30, 16, 2, -12, -26, -40, -54), a midline sagittal (x = 0) and coronal slice at the level of the cerebellum (y =-52) (Montreal Neurological Institute space). Parcellated results shown in combined cerebral surface reconstructions with subcortical standard space in 6 slices (z = 16, 2, -12, -26, -40, -54), consistent with main figures. Laterality follows radiological convention (the right side of the image corresponds to the right side of the brain). Full numerical results underlying the figures are provided in the Supplementary Dataset.

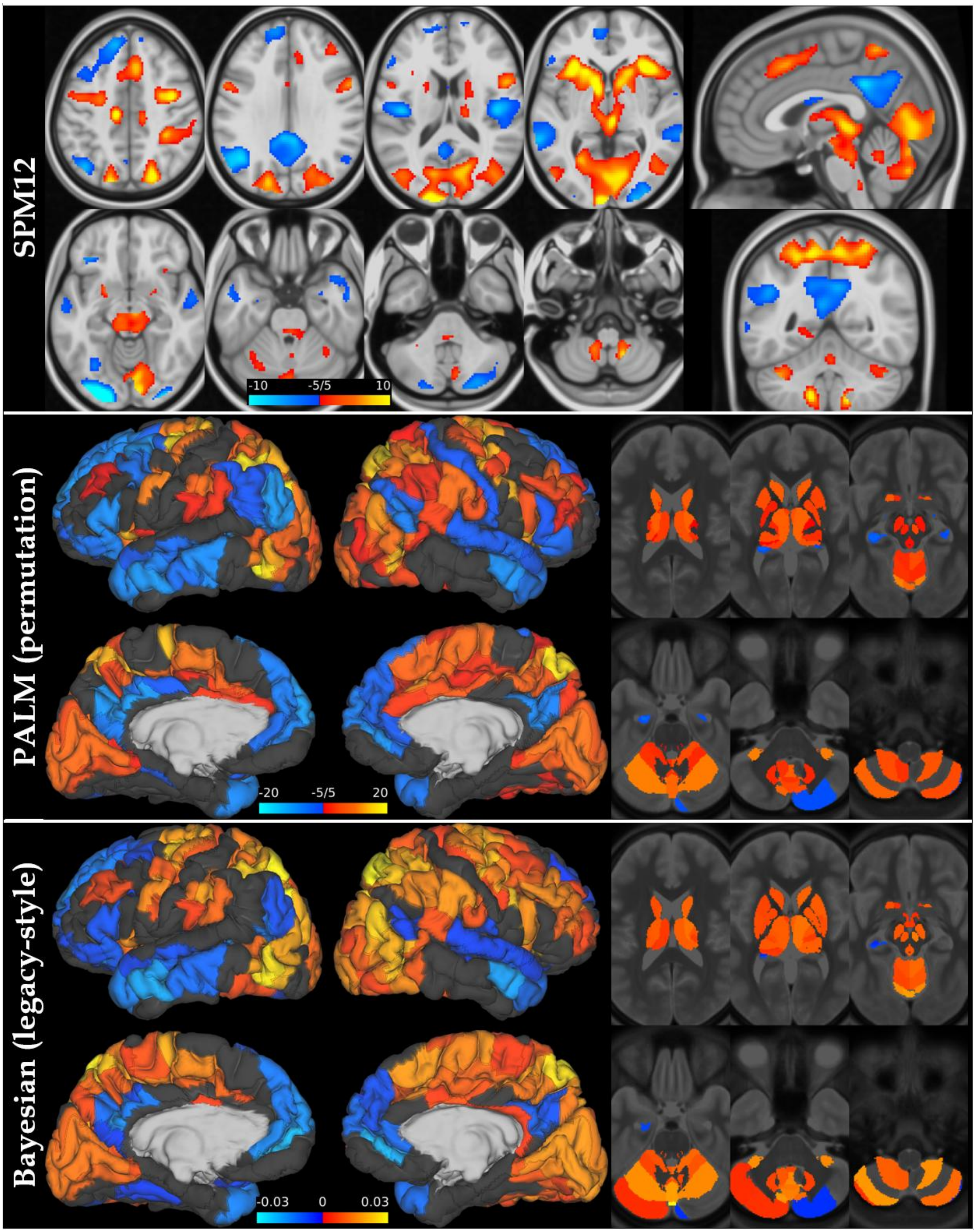

SPM12
-10
-5/5
10
PALM (permutation)
-20
-5/5
20
Bayesian (legacy-style)
-0.03
0
0.03

# Supplementary Tables

**Supplementary Table 1: Overarching anatomical segments used to define parcel groupings for clique-based hierarchies in the second-level Bayesian models.** All parcels within each segment were partially pooled via clique-level effects with separately modelled parcel-level deviations.

| | Partition Segment – Clique | Parcel no. | Category | Source Atlas |
|---|---|---|---|---|
| 1 | Primary Visual Cortex | 2 | Visual/ Occipital Cortex | Human Connectome Project Multimodal Parcellation Atlas *(Glasser et al. 2016)* |
| 2 | MT+ Complex and Neighbouring Visual Areas | 18 | | |
| 3 | Dorsal Stream Visual Cortex | 12 | | |
| 4 | Early Visual Cortex | 6 | | |
| 5 | Ventral Stream Visual Cortex | 14 | | |
| 6 | Somatosensory and Motor Cortex | 10 | Sensori-motor Cortex | |
| 7 | Premotor Cortex | 14 | | |
| 8 | Posterior Opercular Cortex | 12 | | |
| 9 | Paracentral Lobular and Mid Cingulate Cortex | 16 | | |
| 10 | Early Auditory Cortex | 10 | Auditory Cortex | |
| 11 | Auditory Association Cortex | 16 | | |
| 12 | Frontal Opercular and Insular Cortex | 26 | | |
| 13 | Medial Temporal | 14 | Temporal Cortex | |
| 14 | Lateral Temporal Cortex | 18 | | |
| 15 | Temporal-Parietal-Occipital Junction | 10 | Parietal Cortex | |
| 16 | Superior Parietal and IPS | 20 | | |
| 17 | Inferior Parietal Cortex | 20 | | |
| 18 | Posterior Cingulate Cortex | 28 | | |
| 19 | Anterior Cingulate and Medial Prefrontal Cortex | 30 | Frontal Cortex | |
| 20 | Orbital and Polar Frontal Cortex | 22 | | |
| 21 | Inferior Frontal Cortex | 16 | | |
| 22 | Dorsolateral Prefrontal Cortex | 26 | | |
| 23 | Thalamus | 14 | Dience-phalon | Oxford Thalamic Connectivity Probability Atlas *(Behrens et al. 2003)* |
| 24 | Hypothalamus | 4 | | Hypothalamic atlas *(Spindler et al. 2020)* |
| 25 | Striatum | 14 | Basal Ganglia | Oxford-Imanova Striatal Atlas *(Tziortzi et al. 2014)* |
| 26 | Pallidum | 4 | | ATAG for middle aged, elderly *(Keuken et al. 2017)* |
| 27 | Basal Ganglia-Associated Nuclei | 4 | | |
| 28 | Brainstem – Monoaminergic and Arousal Nuclei | 3 | Brainstem | Harvard Ascending Arousal Network Atlas *(Edlow et al. 2012)* |
| 29 | Brainstem – Reticular and Sensorimotor Integration Nuclei | 7 | | |
| 30 | Amygdala | 10 | Limbic System | High-resolution atlas of amygdala *(Saygin et al. 2017)* |
| 31 | Hippocampus | 10 | | In Vivo High-Resolution Atlas of the Hippocampus and Subfields *(Winterburn et al. 2013)* |
| 32 | Cerebellar Cortex – Anterior Sensorimotor | 4 | Cerebellum | Probabilistic structural cerebellar atlas *(Dietrichsen et al. 2009)* |
| 33 | Cerebellar Cortex – VI (Transitional) | 3 | | |
| 34 | Cerebellar Cortex – Executive/Associative | 8 | | |
| 35 | Cerebellar Cortex – Sensorimotor VIII | 6 | | |
| 36 | Cerebellar Cortex – Posterior (IX–X) | 6 | | |
| 37 | Cerebellar Deep Nuclei | 4 | | |

**Supplementary Table 2: Convergence diagnostics and sampler behaviour of second-level Bayesian models** for the main effects of interest. $\hat{R}$ denotes the Gelman–Rubin convergence diagnostic; ESS effective sample size. Bulk ESS and tail ESS provide summaries of sampling efficiency for the bulk of the posterior distribution and for tail quantities (e.g. credible interval endpoints), respectively. Worst-case values across the main effects of interest are reported (minimum ESS; maximum $\hat{R}$).

| Model | Maximal $\hat{R}$ | Minimal bulk ESS | Minimal tail ESS | Divergent transitions | Tree-depth saturations (n) |
|---|---|---|---|---|---|
| **Main task-fMRI model** | 1.009 | 2,261 | 1,328 | 0 | 0 |
| **Microstructural group-level comparison** | 1.005 | 2,119 | 1,711 | 0 | 0 |

**Supplementary Table 3: Results of second level task-related activation effects for motor prediction** (shooting–cross) **and error modulation (**within the same contrast). Corresponding spatial maps provided in the Figure 3. Reported over larger anatomical partitions (cliques, see also Supplementary Table 1) separately for control-group mean effect and expert–control difference. Values provided as posterior medians with 95% credible intervals (CrI) (multiplied by $10^3$ for numerical clarity) and posterior probability that the effect has the reported sign (P(sign)). Arrows indicate effects whose 95% CrI excludes zero (↑ positive, ↓ negative); effects with CrI spanning zero shown in grey. The ordering of cliques follows anatomical hierarchies across cortical, subcortical and cerebellar systems. Full numerical parcellated results are provided in the Supplementary Dataset.

| | Anatomical Partition Segment (clique) | Control-Group Mean Effect of Speed | | | | Expert-Control Difference in Speed | | | |
|---|---|---|---|---|---|---|---|---|---|
| | | Motor Prediction | | Error modulation | | Motor Prediction | | Error modulation | |
| | | median [95% CrI] | P(sign) | median [95% CrI] | P(sign) | median [95% CrI] | P(sign) | median [95% CrI] | P(sign) |
| Visual/Occipit. Cortex | **Primary Visual Cortex** | ↑ 5.6 [1.6,9.5] | 0.998 | ↓ -8.8 [-12.2,-5.2] | 1.000 | ↓ -4.4 [-7.8,-0.6] | 0.989 | 3.0 [-1.5,7.3] | 0.900 |
| | MT+ Complex and Neighbouring Visual Areas | ↑ 1.6 [0.3,2.9] | 0.992 | ↓ -6.7 [-7.7,-5.7] | 1.000 | ↓ -1.6 [-2.8,-0.4] | 0.996 | -0.1 [-1.4,1.2] | 0.557 |
| | **Dorsal Stream Visual Cortex** | ↑ 1.7 [0.2,3.4] | 0.986 | ↓ -6.6 [-7.8,-5.4] | 1.000 | ↓ -2.7 [-4.2,-1.3] | 1.000 | 0.4 [-1.2,2.0] | 0.681 |
| | Early Visual Cortex | ↑ 5.8 [3.4,8.1] | 1.000 | ↓ -9.3 [-11.1,-7.6] | 1.000 | ↓ -4.3 [-6.4,-2.3] | 1.000 | ↑ 2.7 [0.5,4.9] | 0.994 |
| | **Ventral Stream Visual Cortex** | ↑ 6.3 [4.8,7.7] | 1.000 | ↓ -7.7 [-8.8,-6.6] | 1.000 | ↓ -3.6 [-5.0,-2.2] | 1.000 | 1.3 [-0.2,2.8] | 0.960 |
| Sensori-motor cort. | Somatosensory and Motor Cortex | ↑ 2.1 [0.4,3.9] | 0.989 | ↓ -5.2 [-6.5,-3.9] | 1.000 | -1.3 [-2.9,0.4] | 0.935 | -0.8 [-2.4,0.9] | 0.814 |
| | **Premotor Cortex** | -1.1 [-2.5,0.5] | 0.916 | ↓ -6.0 [-7.1,-4.8] | 1.000 | 0.1 [-1.3,1.5] | 0.566 | 0.3 [-1.2,1.7] | 0.644 |
| | Posterior Operculum | ↑ 2.0 [0.4,3.5] | 0.994 | ↓ -6.6 [-7.9,-5.4] | 1.000 | ↓ -1.6 [-3.0,-0.3] | 0.991 | -0.1 [-1.7,1.5] | 0.558 |
| | **Paracentral Lobular and Mid Cingulate** | -0.3 [-1.6,1.1] | 0.668 | ↓ -6.2 [-7.3,-5.2] | 1.000 | 0.0 [-1.3,1.3] | 0.508 | 0.4 [-0.9,1.7] | 0.730 |
| Auditory Cortex | Early Auditory Cortex | ↑ 2.0 [0.3,3.7] | 0.989 | ↓ -6.4 [-7.8,-5.1] | 1.000 | -1.2 [-2.7,0.2] | 0.950 | 0.8 [-0.9,2.6] | 0.824 |
| | **Auditory Association Cortex** | ↑ 5.4 [4.1,6.8] | 1.000 | ↓ -5.0 [-6.1,-4.0] | 1.000 | ↓ -3.2 [-4.5,-2.0] | 1.000 | ↑ 1.9 [0.5,3.2] | 0.998 |
| | Frontal Opercular and Insular Cortex | 0.0 [-1.1,1.1] | 0.502 | ↓ -7.0 [-7.8,-6.2] | 1.000 | ↓ -1.1 [-2.0,-0.1] | 0.984 | ↑ 1.2 [0.1,2.3] | 0.988 |
| Tem. Cort. | **Medial Temporal** | ↑ 3.9 [2.1,5.6] | 1.000 | ↓ -4.7 [-6.1,-3.2] | 1.000 | ↓ -2.0 [-3.6,-0.4] | 0.992 | -0.3 [-2.0,1.5] | 0.618 |
| | Lateral Temporal Cortex | ↑ 4.6 [2.9,6.2] | 1.000 | ↓ -4.2 [-5.5,-3.0] | 1.000 | ↓ -2.5 [-3.9,-1.1] | 0.999 | 0.0 [-1.6,1.7] | 0.522 |
| Parietal Cortex | **Temporal-Parietal-Occipital Junction** | 0.7 [-0.9,2.4] | 0.797 | ↓ -7.4 [-8.7,-6.0] | 1.000 | -1.0 [-2.4,0.6] | 0.902 | 1.2 [-0.5,3.0] | 0.923 |
| | Superior Parietal and IPS | ↓ -1.9 [-3.2,-0.6] | 0.999 | ↓ -5.4 [-6.3,-4.4] | 1.000 | 0.6 [-0.7,1.7] | 0.815 | 0.5 [-0.8,1.7] | 0.772 |
| | **Inferior Parietal Cortex** | 0.2 [-1.0,1.4] | 0.628 | ↓ -6.0 [-7.0,-4.9] | 1.000 | -1.1 [-2.2,0.1] | 0.968 | -0.1 [-1.4,1.2] | 0.557 |
| | Posterior Cingulate | ↑ 4.1 [3.0,5.1] | 1.000 | ↓ -5.7 [-6.5,-4.9] | 1.000 | ↓ -1.8 [-2.8,-0.8] | 1.000 | -0.5 [-1.6,0.5] | 0.829 |
| Frontal Cortex | **Anterior Cingulate** | ↑ 2.6 [1.5,3.7] | 1.000 | ↓ -5.2 [-6.1,-4.3] | 1.000 | -0.8 [-1.9,0.2] | 0.934 | ↑ 1.3 [0.2,2.4] | 0.988 |
| | Orbital and Polar Frontal Cortex | ↑ 3.5 [2.1,4.9] | 1.000 | ↓ -5.7 [-6.7,-4.6] | 1.000 | -0.2 [-1.5,1.0] | 0.643 | ↑ 1.9 [0.6,3.3] | 0.996 |
| | **Inferior Frontal Cortex** | ↑ 1.6 [0.2,3.1] | 0.989 | ↓ -7.4 [-8.4,-6.4] | 1.000 | ↓ -1.4 [-2.8,-0.2] | 0.987 | ↑ 1.9 [0.6,3.3] | 0.999 |
| | Dorsolateral Prefrontal Cortex | ↑ 2.2 [1.1,3.3] | 1.000 | ↓ -5.1 [-5.9,-4.2] | 1.000 | -0.5 [-1.5,0.6] | 0.817 | 1.0 [-0.1,2.1] | 0.956 |
| Dien ceph. | **Thalamus** | 0.9 [-0.6,2.3] | 0.871 | ↓ -5.7 [-6.9,-4.6] | 1.000 | 0.1 [-1.3,1.5] | 0.553 | 0.4 [-1.1,1.9] | 0.711 |
| | Hypothalamus | 0.6 [-2.1,3.5] | 0.672 | ↓ -5.9 [-8.1,-3.7] | 1.000 | 1.0 [-1.5,3.5] | 0.774 | 2.6 [-0.3,5.4] | 0.959 |
| Basal Ganglia | **Striatum** | -1.0 [-2.5,0.4] | 0.916 | ↓ -6.1 [-7.2,-5.0] | 1.000 | 1.3 [-0.1,2.7] | 0.968 | 0.7 [-0.8,2.0] | 0.812 |
| | Pallidum | -0.2 [-2.9,2.4] | 0.575 | ↓ -6.0 [-8.1,-3.8] | 1.000 | 0.4 [-2.0,2.7] | 0.611 | 1.0 [-1.7,3.7] | 0.762 |
| | **Basal Ganglia-Associated Nuclei** | 1.5 [-1.2,4.3] | 0.854 | ↓ -6.4 [-8.6,-4.2] | 1.000 | ↓ -3.6 [-5.9,-1.1] | 0.999 | 0.4 [-2.5,3.4] | 0.611 |
| | Brainstem – Reticular and Sensorimotor Nuclei | -0.7 [-3.3,1.7] | 0.715 | ↓ -5.3 [-7.5,-3.1] | 1.000 | 0.2 [-2.3,2.4] | 0.550 | -0.2 [-3.0,2.7] | 0.542 |
| Limb. Syst. | **Amygdala** | ↑ 5.3 [3.0,7.7] | 1.000 | -0.4 [-2.4,1.5] | 0.677 | -1.3 [-3.3,0.7] | 0.893 | ↓ -4.1 [-6.6,-1.6] | 0.998 |
| | Hippocampus | ↑ 4.8 [3.0,6.5] | 1.000 | ↓ -2.3 [-3.6,-1.0] | 0.999 | ↓ -2.0 [-3.5,-0.4] | 0.991 | ↓ -2.3 [-4.1,-0.6] | 0.993 |
| Cerebellum | **Cerebellum – Anterior Sensorimotor** | -0.5 [-3.3,2.3] | 0.628 | ↓ -4.9 [-7.0,-2.9] | 1.000 | 1.6 [-0.9,4.2] | 0.885 | 1.1 [-1.8,3.7] | 0.757 |
| | Cerebellum – VI (Transitional) | 0.1 [-3.6,3.7] | 0.521 | ↓ -7.0 [-9.7,-4.1] | 1.000 | 0.4 [-3.2,4.1] | 0.594 | 2.2 [-1.5,5.7] | 0.883 |
| | **Cerebellum – Executive/Associative** | 0.2 [-1.7,2.1] | 0.574 | ↓ -5.3 [-6.8,-3.8] | 1.000 | 0.4 [-1.3,2.1] | 0.701 | 1.8 [-0.1,3.7] | 0.972 |
| | Cerebellum – Sensorimotor VIII | -0.3 [-2.5,1.8] | 0.615 | ↓ -3.7 [-5.5,-2.1] | 1.000 | 1.2 [-0.8,3.2] | 0.884 | 0.0 [-2.2,2.2] | 0.511 |
| | **Cerebellum – Posterior (IX–X)** | -0.6 [-2.7,1.7] | 0.695 | ↓ -5.3 [-7.0,-3.6] | 1.000 | ↑ 2.0 [0.0,3.8] | 0.976 | 1.7 [-0.5,4.0] | 0.932 |
| | Cerebellar Deep Nuclei | ↑ 4.0 [1.0,6.9] | 0.996 | ↓ -4.3 [-6.5,-2.3] | 1.000 | -1.6 [-4.4,1.1] | 0.876 | 2.0 [-0.8,4.6] | 0.921 |

**Supplementary Table 4: Microstructural inter-group comparison.** Corresponding spatial maps provided in the Figure 4. Reported over larger anatomical partitions (cliques, see also Supplementary Table 1) separately for individual microstructural metrics – Neurite Density Index (NDI), Orientation Dispersion Index (ODI) and adiabatic $T_1\rho$ relaxation constant (analysed only in subcortical structures due to the field of view utilised in the MRI acquisition). Values provided as metric medians [$10^{th}$–$90^{th}$ percentiles] for control and expert groups separately, followed by group differences expressed as posterior medians with 95% credible intervals (CrI) and posterior probability that the effect has the reported sign (P(sign)). Arrows indicate effects whose 95% CrI excludes zero (↑ positive, ↓ negative); effects with CrI spanning zero shown in grey. The ordering of cliques follows anatomical hierarchies across cortical, subcortical and cerebellar systems. Full numerical parcellated results are provided in the Supplementary Dataset.

| Anatomical Partition Segment (clique) | | Neurite Density Index [*unitless*] | | | | Orientation Dispersion Index [*unitless*] | | | | Adiabatic $T_1\rho$ relaxation constants [*ms*] | | | |
|---|---|---|---|---|---|---|---|---|---|---|---|---|---|
| | | Controls | Experts | median [95% CrI] | P(sign) | Controls | Experts | median [95% CrI] | P(sign) | Controls | Experts | median [95% CrI] | P(sign) |
| Visual/Occipit. Cortex | **Primary Visual Cortex** | 0.403 [0.387,0.422] | 0.404 [0.386,0.420] | -0.09 [-0.36,0.16] | 0.754 | 0.614 [0.588,0.642] | 0.629 [0.598,0.649] | ↑ 0.43 [0.15,0.72] | 0.999 | | | | |
| | MT+ Complex and Neighbouring Visual Areas | 0.400 [0.383,0.422] | 0.406 [0.389,0.427] | ↑ 0.37 [0.29,0.46] | 1.000 | 0.598 [0.556,0.647] | 0.601 [0.557,0.647] | 0.07 [-0.03,0.16] | 0.916 | | | | |
| | **Dorsal Stream Visual Cortex** | 0.398 [0.375,0.419] | 0.404 [0.383,0.426] | ↑ 0.44 [0.33,0.55] | 1.000 | 0.591 [0.544,0.638] | 0.592 [0.550,0.633] | 0.04 [-0.08,0.17] | 0.738 | | | | |
| | Early Visual Cortex | 0.407 [0.391,0.427] | 0.413 [0.398,0.430] | ↑ 0.17 [0.01,0.33] | 0.981 | 0.611 [0.583,0.636] | 0.613 [0.590,0.637] | ↑ 0.17 [0.00,0.34] | 0.978 | | | | |
| | **Ventral Stream Visual Cortex** | 0.416 [0.392,0.437] | 0.420 [0.399,0.440] | ↑ 0.26 [0.16,0.36] | 1.000 | 0.603 [0.558,0.647] | 0.601 [0.553,0.649] | -0.04 [-0.14,0.07] | 0.746 | | | | |
| Sensori-motor cort. | Somatosensory and Motor Cortex | 0.416 [0.383,0.459] | 0.419 [0.388,0.454] | ↑ 0.22 [0.10,0.35] | 1.000 | 0.570 [0.453,0.616] | 0.575 [0.474,0.617] | ↑ 0.23 [0.11,0.36] | 1.000 | | | | |
| | **Premotor Cortex** | 0.405 [0.378,0.434] | 0.409 [0.384,0.440] | ↑ 0.31 [0.20,0.41] | 1.000 | 0.553 [0.506,0.596] | 0.559 [0.516,0.598] | ↑ 0.23 [0.12,0.34] | 1.000 | | | | |
| | Posterior Operculum | 0.397 [0.377,0.419] | 0.403 [0.381,0.428] | ↑ 0.46 [0.34,0.57] | 1.000 | 0.569 [0.524,0.617] | 0.569 [0.523,0.614] | -0.01 [-0.14,0.10] | 0.591 | | | | |
| | **Paracentral Lobular and Mid Cingulate** | 0.407 [0.378,0.433] | 0.412 [0.383,0.440] | ↑ 0.33 [0.24,0.42] | 1.000 | 0.574 [0.520,0.624] | 0.578 [0.528,0.626] | ↑ 0.18 [0.07,0.28] | 1.000 | | | | |
| Auditory Cortex | Early Auditory Cortex | 0.424 [0.402,0.449] | 0.431 [0.411,0.455] | ↑ 0.46 [0.34,0.58] | 1.000 | 0.606 [0.549,0.662] | 0.608 [0.560,0.662] | 0.09 [-0.04,0.22] | 0.909 | | | | |
| | **Auditory Association Cortex** | 0.394 [0.375,0.413] | 0.398 [0.379,0.416] | ↑ 0.32 [0.22,0.41] | 1.000 | 0.580 [0.521,0.629] | 0.579 [0.522,0.621] | -0.05 [-0.16,0.05] | 0.857 | | | | |
| | Frontal Opercular and Insular Cortex | 0.396 [0.373,0.419] | 0.400 [0.377,0.423] | ↑ 0.37 [0.29,0.44] | 1.000 | 0.528 [0.438,0.590] | 0.535 [0.435,0.589] | 0.03 [-0.06,0.11] | 0.737 | | | | |
| Tem. Cort. | **Medial Temporal** | 0.422 [0.399,0.468] | 0.424 [0.403,0.481] | ↑ 0.23 [0.11,0.36] | 1.000 | 0.576 [0.465,0.639] | 0.571 [0.463,0.640] | ↓ -0.13 [-0.26,0.00] | 0.977 | | | | |
| | Lateral Temporal Cortex | 0.392 [0.375,0.439] | 0.397 [0.377,0.430] | ↑ 0.14 [0.04,0.25] | 0.998 | 0.578 [0.530,0.622] | 0.579 [0.531,0.628] | 0.04 [-0.07,0.17] | 0.771 | | | | |
| Parietal Cortex | **Temporal-Parietal-Occipital Junction** | 0.397 [0.380,0.414] | 0.404 [0.388,0.421] | ↑ 0.59 [0.47,0.70] | 1.000 | 0.582 [0.543,0.622] | 0.587 [0.546,0.631] | ↑ 0.15 [0.03,0.28] | 0.991 | | | | |
| | Superior Parietal and IPS | 0.382 [0.363,0.407] | 0.390 [0.366,0.414] | ↑ 0.42 [0.34,0.50] | 1.000 | 0.569 [0.514,0.616] | 0.577 [0.530,0.619] | ↑ 0.29 [0.20,0.39] | 1.000 | | | | |
| | **Inferior Parietal Cortex** | 0.391 [0.373,0.409] | 0.396 [0.379,0.415] | ↑ 0.49 [0.40,0.57] | 1.000 | 0.572 [0.532,0.608] | 0.575 [0.538,0.610] | ↑ 0.11 [0.02,0.20] | 0.991 | | | | |
| | Posterior Cingulate | 0.407 [0.387,0.430] | 0.414 [0.394,0.436] | ↑ 0.55 [0.47,0.62] | 1.000 | 0.575 [0.533,0.623] | 0.573 [0.529,0.617] | ↓ -0.08 [-0.16,0.00] | 0.977 | | | | |
| Frontal Cortex | **Anterior Cingulate** | 0.397 [0.377,0.422] | 0.399 [0.378,0.425] | ↑ 0.19 [0.11,0.27] | 1.000 | 0.577 [0.525,0.645] | 0.573 [0.520,0.642] | -0.02 [-0.10,0.06] | 0.718 | | | | |
| | Orbital and Polar Frontal Cortex | 0.396 [0.361,0.439] | 0.398 [0.366,0.432] | 0.05 [-0.05,0.14] | 0.832 | 0.591 [0.540,0.632] | 0.592 [0.541,0.635] | 0.05 [-0.06,0.16] | 0.828 | | | | |
| | **Inferior Frontal Cortex** | 0.393 [0.374,0.423] | 0.398 [0.378,0.426] | ↑ 0.27 [0.17,0.37] | 1.000 | 0.576 [0.531,0.618] | 0.581 [0.533,0.623] | ↑ 0.15 [0.05,0.26] | 0.998 | | | | |
| | Dorsolateral Prefrontal Cortex | 0.390 [0.367,0.411] | 0.391 [0.372,0.415] | ↑ 0.21 [0.14,0.28] | 1.000 | 0.567 [0.523,0.604] | 0.572 [0.529,0.611] | ↑ 0.22 [0.14,0.30] | 1.000 | | | | |
| Dienceph. | **Thalamus** | 0.621 [0.564,0.688] | 0.624 [0.570,0.699] | ↑ 0.22 [0.12,0.32] | 1.000 | 0.329 [0.279,0.389] | 0.326 [0.274,0.388] | -0.08 [-0.19,0.04] | 0.903 | 114.9 [102.8,133.0] | 115.5 [105.4,133.4] | 0.10 [0.00,0.19] | 0.974 |
| | Hypothalamus | 0.499 [0.419,0.629] | 0.504 [0.431,0.662] | ↑ 0.31 [0.12,0.50] | 0.998 | 0.435 [0.352,0.525] | 0.430 [0.357,0.536] | 0.05 [-0.15,0.25] | 0.691 | 156.7 [127.8,181.2] | 155.5 [127.1,180.6] | 0.12 [-0.06,0.29] | 0.903 |
| Basal Ganglia | **Striatum** | 0.503 [0.469,0.549] | 0.511 [0.473,0.563] | ↑ 0.39 [0.29,0.49] | 1.000 | 0.607 [0.532,0.667] | 0.610 [0.525,0.669] | 0.10 [-0.02,0.20] | 0.952 | 121.1 [108.4,134.9] | 121.9 [109.6,137.0] | ↑ 0.11 [0.02,0.21] | 0.990 |
| | Pallidum | 0.902 [0.790,0.979] | 0.910 [0.808,0.988] | 0.15 [-0.04,0.34] | 0.942 | 0.504 [0.391,0.563] | 0.514 [0.401,0.573] | 0.11 [-0.10,0.32] | 0.849 | 100.1 [90.9,111.1] | 100.1 [93.8,108.8] | 0.02 [-0.14,0.19] | 0.601 |
| | **Basal Ganglia-Associated Nuclei** | 0.874 [0.753,0.946] | 0.892 [0.766,0.965] | 0.16 [-0.03,0.34] | 0.952 | 0.331 [0.263,0.399] | 0.331 [0.260,0.399] | 0.15 [-0.06,0.35] | 0.920 | 102.1 [87.3,115.8] | 103.0 [88.9,115.3] | 0.06 [-0.12,0.23] | 0.731 |
| | Brainstem – Reticular and Sensorimotor Nuclei | 0.739 [0.452,0.871] | 0.746 [0.458,0.879] | 0.10 [-0.08,0.27] | 0.868 | 0.343 [0.175,0.445] | 0.344 [0.180,0.444] | 0.15 [-0.04,0.33] | 0.938 | 109.7 [93.6,140.5] | 108.9 [96.9,140.5] | 0.00 [-0.16,0.17] | 0.506 |
| Limb. Syst. | **Amygdala** | 0.417 [0.390,0.459] | 0.416 [0.390,0.465] | -0.04 [-0.19,0.12] | 0.671 | 0.525 [0.441,0.597] | 0.513 [0.431,0.578] | ↓ -0.38 [-0.54,-0.21] | 1.000 | 141.1 [127.7,159.9] | 141.5 [131.4,162.9] | ↑ 0.17 [0.03,0.32] | 0.992 |
| | Hippocampus | 0.425 [0.388,0.498] | 0.421 [0.389,0.484] | -0.05 [-0.17,0.07] | 0.812 | 0.557 [0.382,0.640] | 0.551 [0.390,0.636] | -0.08 [-0.21,0.06] | 0.885 | 130.8 [113.9,148.4] | 132.3 [116.4,148.0] | ↑ 0.17 [0.05,0.28] | 0.998 |
| Cerebellum | **Cerebellum – Anterior Sensorimotor** | 0.555 [0.507,0.607] | 0.562 [0.514,0.610] | ↑ 0.20 [0.01,0.39] | 0.982 | 0.518 [0.485,0.552] | 0.515 [0.486,0.553] | -0.06 [-0.26,0.14] | 0.737 | 139.5 [126.8,155.5] | 137.8 [127.1,150.4] | ↓ -0.19 [-0.37,-0.02] | 0.984 |
| | Cerebellum – VI (Transitional) | 0.516 [0.450,0.553] | 0.528 [0.468,0.562] | ↑ 0.34 [0.12,0.55] | 0.998 | 0.504 [0.456,0.567] | 0.501 [0.458,0.573] | 0.02 [-0.22,0.26] | 0.550 | 136.7 [125.9,151.0] | 134.4 [128.0,145.2] | -0.15 [-0.35,0.06] | 0.925 |
| | **Cerebellum – Executive/Associative** | 0.520 [0.469,0.567] | 0.524 [0.482,0.565] | ↑ 0.14 [0.01,0.27] | 0.979 | 0.473 [0.438,0.528] | 0.466 [0.430,0.516] | ↓ -0.28 [-0.42,-0.13] | 1.000 | 137.2 [125.2,151.5] | 136.2 [125.0,146.2] | ↓ -0.19 [-0.31,-0.06] | 0.997 |
| | Cerebellum – Sensorimotor VIII | 0.574 [0.497,0.656] | 0.588 [0.510,0.658] | ↑ 0.35 [0.20,0.51] | 1.000 | 0.518 [0.476,0.566] | 0.509 [0.471,0.550] | ↓ -0.18 [-0.35,-0.01] | 0.984 | 142.5 [127.3,166.0] | 139.3 [127.5,160.3] | ↓ -0.18 [-0.32,-0.04] | 0.990 |
| | **Cerebellum – Posterior (IX–X)** | 0.681 [0.610,0.765] | 0.693 [0.608,0.768] | ↑ 0.19 [0.03,0.35] | 0.992 | 0.463 [0.367,0.589] | 0.462 [0.360,0.571] | ↓ -0.27 [-0.44,-0.10] | 1.000 | 147.1 [108.4,181.8] | 145.9 [108.4,182.9] | -0.01 [-0.16,0.14] | 0.532 |
| | Cerebellar Deep Nuclei | 0.503 [0.405,0.679] | 0.518 [0.410,0.695] | ↑ 0.27 [0.08,0.46] | 0.998 | 0.384 [0.301,0.557] | 0.379 [0.318,0.537] | -0.03 [-0.22,0.19] | 0.606 | 124.4 [110.4,140.9] | 125.9 [110.9,141.6] | 0.07 [-0.11,0.25] | 0.794 |

# Supplementary references


Filip, Pavel, Cécile Gallea, Stéphane Lehéricy, et al. 2017. "Disruption in Cerebellar and Basal Ganglia Networks during a Visuospatial Task in Cervical Dystonia." *Movement Disorders: Official Journal of the Movement Disorder Society* 32 (5): 757–68. https://doi.org/10.1002/mds.26930.

Friston, K. J., A. P. Holmes, K. J. Worsley, J. P. Poline, C. D. Frith, and R. S. J. Frackowiak. 1994. "Statistical Parametric Maps in Functional Imaging: A General Linear Approach." *Human Brain Mapping* 2 (4): 189–210. https://doi.org/10.1002/hbm.460020402.

Gelman, Andrew, John B. Carlin, Hal S. Stern, David B. Dunson, Akti Vehtari, and Donald B. Rubin. 2013. *Bayesian Data Analysis*. Third. Chapman & Hall/CRC Texts in Statistical Science Series. CRC. https://stat.columbia.edu/~gelman/book/.

Gelman, Andrew, Jennifer Hill, and Masanao Yajima. 2012. "Why We (Usually) Don't Have to Worry About Multiple Comparisons." *Journal of Research on Educational Effectiveness* 5 (2): 189–211. https://doi.org/10.1080/19345747.2011.618213.

Winkler, Anderson M., Gerard R. Ridgway, Matthew A. Webster, Stephen M. Smith, and Thomas E. Nichols. 2014. "Permutation Inference for the General Linear Model." *NeuroImage* 92 (May): 381–97. https://doi.org/10.1016/j.neuroimage.2014.01.060.
